# Overstated association between adolescent physical fitness and adulthood depression risk due to familial factors

**DOI:** 10.1111/joim.20109

**Published:** 2025-07-09

**Authors:** Marcel Ballin, Örjan Ekblom, Anna Nordström, Viktor H. Ahlqvist, Peter Nordström

**Affiliations:** ^1^ Department of Public Health and Caring Sciences Clinical Geriatrics Uppsala University Uppsala Sweden; ^2^ Department of Physical Activity and Health The Swedish School of Sport and Health Sciences Stockholm Sweden; ^3^ Department of Medical Sciences Rehabilitation Medicine Uppsala University Uppsala Sweden; ^4^ School of Sport Sciences UiT The Arctic University of Norway Tromsø Norway; ^5^ Department of Biomedicine Aarhus University Aarhus Denmark; ^6^ Institute of Environmental Medicine, Karolinska Institutet Stockholm Sweden

**Keywords:** depressive disorders, epidemiology, physical activity, prevention, public health

## Abstract

**Objective:**

Examine the association between adolescent cardiorespiratory fitness and future risk of depression and dispensation of antidepressants, including the role of familial confounding.

**Methods:**

A cohort study with sibling‐comparisons based on Swedish men who participated in mandatory military conscription examinations from 1972 to 1995. The exposure was cardiorespiratory fitness estimated using a maximal ergometer bicycle test. The outcomes were depression diagnosis in specialized outpatient or inpatient care and dispensation of antidepressants until 31 December 2023.

**Results:**

A total of 1,013,885 men (mean age 18.3 years), of which 410,198 were full siblings, were followed until a median age of 56.8 years, during which 47,283 were diagnosed with depression and 237,409 were dispensed antidepressants. In cohort analysis, the highest decile of fitness had lower risks of depression (adjusted hazard ratio [HR] 0.54, [95% confidence interval, 0.52, 0.57]) and antidepressants (HR 0.63; 0.62, 0.65) compared to the lowest decile, with differences in the standardized cumulative incidence by age 65 of −3.9% and −12.3%, respectively. In sibling‐comparison analyses accounting for unobserved familial confounders, the associations attenuated for both depression (HR 0.67, 0.59–0.75; incidence difference −2.4%) and antidepressants (HR 0.76, 0.72–0.80; incidence difference −7.2%). Hypothetically shifting everyone to the highest decile of fitness was associated with a preventable fraction of 29.1% for depression and 17.8% for antidepressants in cohort analysis, which attenuated to 17.6% and 10.4% in sibling‐comparisons.

**Conclusions:**

High levels of adolescent cardiorespiratory fitness are associated with lower risks of future depression and antidepressants, but the associations might be overstated due to familial confounding.

## Graphical abstract

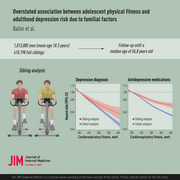

**Abbreviations**
BMI: body mass index
CI: confidence interval
ICD: International Classification of Diseases
HR: hazard ratio
Wmax: watt maximum
VO_2_max: maximal oxygen uptake

## Introduction

The prevalence of depression and other mental health problems remains at a staggering level, presenting a significant global health burden [1, 2]. Although modifiable factors such as physical exercise can play a role in treatment outcomes when combined with usual care [3], attention has also turned to whether exercise could prevent the onset of mental health problems, including depression [4].

Recent studies have found a protective association between cardiorespiratory fitness—a marker of long‐term exercise and genetic predisposition [5, 6]—and incident depression and use of antidepressants [7, 8, 9, 10, 11, 12, 13], with proposed mechanisms, including reduced stress [14], enhanced psychosocial risk factors [15] and effects on neurotrophic processes, monoamine metabolism and inflammation [16]. However, whether these epidemiological associations reflect causal relationships (i.e., increasing fitness will lower the risk of depression) or whether fitness is a marker of a broader, well‐balanced lifestyle that coincides with better mental health remains unclear. This distinction has important implications; if causal, the findings could inform targeted interventions to reduce the incidence of depression. Alternatively, if primarily serving as a marker, the emphasis may need to shift toward assessing fitness for screening purposes. Unfortunately, disentangling a potential causal role of cardiorespiratory fitness in depression etiology has proven challenging because the field relies heavily on observational evidence—much of which lacks an adequate control for key biases, including unobserved familial confounding factors. Moreover, the abundance of studies has focused on middle‐aged individuals, but very few have investigated the role of adolescent cardiorespiratory fitness for long‐term risk of depression [9], which could have public health relevance as fitness levels track from adolescence into adulthood [17].

In this study, we leverage Swedish conscription data on standardized cardiorespiratory fitness assessments of nearly the entire male adolescent population, linked to nationwide healthcare and population registries, to investigate the association between adolescent cardiorespiratory fitness and future risk of incident depression and antidepressant dispensations in over 1 million young men. To distinguish between a screening effect and a putative causal relationship, we compare the standard cohort analysis with estimates derived using sibling‐comparison analysis involving over 400,000 full siblings, thus accounting for all shared behavioural, environmental and genetic factors [18].

## Methods

### Study design

We designed a cohort study by cross‐linking data from Swedish registries using the Personal Identification Number, which is unique for all Swedish residents [19]. The study was approved by the Regional Ethical Review Board in Umeå and later by the Swedish Ethical Review Authority (no. 2010‐113‐31M), who waived the need for informed consent [20]. The study is reported according to the RECORD guidelines [21].

### Databases

The eligible study population was obtained from the Swedish Military Service Conscription Register [22] and was based on all men who participated in military conscription examinations between 1972 and 1995. During this period, conscription around the age of 18 years was mandatory for all Swedish men, with few exemptions (approximately 90% population coverage) [22]. Full siblings were identified using the Multi‐Generation Register, ensuring accurate linkage of familial relationships [23]. Using the National Patient Register [24], we collected data on depression diagnoses from inpatient and specializt outpatient care. Using the Prescribed Drug register, we collected data on antidepressive medications dispensed at pharmacies [25]. Deaths were tracked using the Cause of Death Register [23], and emigration and socioeconomic data were obtained from Statistics Sweden [23]. Overall, these data cover the entire Swedish population and are mandated by law.

### Derivation of study population

A total of 1,249,131 men were conscripted between 1972 and 1995. From these, we excluded 33,645 (2.7%) with missing fitness data, 182,856 (14.6%) with missing covariate data and 18,745 (1.5%) with extreme values (detailed below). This resulted in 1,013,885 individuals being included in the cohort analysis (81.2% retained). Among these, 410,198 were full siblings from 188,946 families and were included in the sibling analysis (Fig. S1).

### Estimation of cardiorespiratory fitness

Cardiorespiratory fitness was estimated at conscription using a maximal ergometer bicycle test according to a standardized procedure [22]. In short, conscripts performed the test following the presentation of normal electrocardiography. The conscript began cycling for 5 min at 60–70 revolutions per minute at a low level of external resistance that was predetermined according to body weight. From thereon, resistance was gradually increased by 25 W/min until exhaustion. The results of the tests were recorded as Watt maximum (Wmax), which served as the exposure in the primary analysis. Wmax has been found to correlate strongly with measured maximal oxygen uptake (gold standard) [26]. We excluded conscripts with extremely low (<100 Wmax) or high (≥999 Wmax) values, as these were considered to likely be due to data entry errors.

### Ascertainment of outcomes

Depression diagnosis and dispensation of antidepressants were studied as two separate outcomes. Diagnosis of depression was defined as the date of first hospitalization or visit in specialized outpatient care in the National Patient Register, recorded from 1997 until 31 December 2023 using the International Classification of Diseases (ICD) 10th revision code F32 in a primary or secondary position. The diagnostic validity of inpatient diagnoses in the National Patient Register is typically high (positive predictive values between 85% and 95% for most diagnoses), although sensitivity is typically lower [24]. The previous validation study did not describe data for depression, but a recent sub‐study found fair‐to‐moderate agreement between depression diagnoses in the National Patient Register and another clinical register [27]. Dispensation of antidepressants was defined as the date of first dispensation, recorded from July 2005 until 31 December 2023 using the Anatomical Therapeutic Chemical code N06A in the Prescribed Drug Register. Dispensation of antidepressants would likely capture both some depression diagnosed in primary care (as primary care is not covered in the National Patient Register) and other milder psychiatric disorders such as anxiety disorders.

### Covariates

From the Swedish Military Service Conscription Register, we collected data on age at conscription, year of conscription, objectively measured body mass index (BMI, kg/m^2^) and results from an IQ‐test, as described previously [22]. Conscripts with extreme BMI values (≤15 and ≥60 kg/m^2^) were excluded. IQ was assessed using a standardized test battery involving subtests covering logical, verbal and visuospatial abilities, as well as a technical test. The scores from each of the individual subtests are summed to a total score. To enhance comparability with studies using other intelligence tests, the total scores were standardized annually to a mean value of 100 with a standard deviation of 15. From Statistics Sweden [28], we obtained data on the socioeconomic status of both mothers and fathers of the conscripts, including information on the highest attained lifetime education and the annual disposable income standardized by birth years into highest achieved quintiles between ages 40 and 50 (hereafter referred to as income categories). The rationale for this approach was that we aimed to capture working‐life income, assuming it to be a better proxy of socioeconomic status than income at the age of conscription. When values for both the mother and father were available, the highest value was retained.

### Statistical analysis

Participants were followed from the date of conscription until the date of depression diagnosis or dispensation of antidepressants (respectively, depending on the outcome under study), emigration, death or end of follow‐up (31 December 2023), whichever came first, using age as the underlying time scale. The associations were modelled using flexible parametric survival models with baseline knots placed at the 5th, 27.5th, 50th, 72.5th and 95th percentiles of the uncensored log survival times [29, 30]. To allow for a nonlinear association, we modelled cardiorespiratory fitness both as deciles and using restricted cubic splines with knots placed at the 5th, 35th, 65th and 95th percentiles [30]. The knot placements were based on Harrell's recommendations [30]. In contrast to Cox regression, the flexible parametric model directly estimates the baseline hazard, thereby enabling the computation of both hazard ratios (HRs) and absolute risk measures [29]. This enabled us to also compute the standardized cumulative incidences (1—Survival) by 65 years of age. Additionally, we estimated the preventable fraction of depression and antidepressants associated with a set of hypothetical interventions of population‐levels of fitness, including a ‘moderate’ (shifting everyone in the bottom four deciles to the fifth decile) and ‘extreme’ (shifting everyone to the 10th decile) intervention [31]. We estimated an unadjusted model followed by a model adjusted for covariates which, based on existing literature and clinical expertise, were assumed to be causally related to both the exposure and the outcome (Fig. S2). These included age at conscription (continuous), year of conscription (1972, 1973–1977, 1978–1982, 1983–1987, 1988–1992 and 1993–1995), BMI (continuous and quadratic term), IQ (continuous), parental education (compulsory school ≤9 years, secondary education, post‐secondary education <3 years, post‐secondary education ≥3 years) and parental income (five categories).

In exploratory analyses, we examined whether BMI acted as an effect modifier by incorporating product terms between overweight status (<25 or ≥25 kg/m^2^) and fitness deciles into the aforementioned model. We then computed marginal HRs across overweight status for the total population while allowing for effect modification, and we post‐estimated HRs within strata of overweight status.

#### Sibling‐comparison analysis

For the sibling‐comparison analysis, we extended the aforementioned model to a marginalized between‐within model with robust (sandwich) standard errors (to account for the clustered nature of the data), which enabled further control for unobserved shared confounders (including shared environmental and behavioural factors and 50% genetic factors [i.e., the proportion shared between full siblings]) [18, 32]. The between–within model isolates the individual‐level variation (within effect) from the family‐level variation (between effect) by including a term for the exposure/covariate and a term for its family average [18, 32]. All analyses were performed using Stata MP version 16.1.

#### Sensitivity analyses

We performed a series of sensitivity analyses. First, to test whether differences in estimates in sibling analysis as compared to cohort analysis were more likely to be due to selection bias into the ‘sibling cohort’ rather than control for unobserved shared confounders, the standard analysis (i.e., not controlling for shared confounders) was replicated in the sibling cohort [18]. Second, because ICD‐10 was not implemented until 1997, meaning that conscripts from the earlier cohorts could have been followed for many years before having the possibility to become diagnosed with depression, we repeated the analyses restricted to those conscripted in the year 1985 or later. Third, to explore the potential for residual confounding from the categorical covariate year of conscription, we (i) repeated the analyses after modelling year of conscription using restricted cubic splines with knots at the 5th, 35th, 65th and 95th percentiles, and (ii) we performed a stratified Cox regression conditioned on year of conscription. Fourth, to relax the proportional hazards assumption, we repeated the analyses and computed the standardized incidences by age 65 after allowing the effect of fitness to vary across time, using an interaction between a restricted cubic spline with three degrees of freedom of the follow‐up time (Centiles 33 and 67 of the distribution of the uncensored log survival times) and the fitness deciles [29]. Fifth, we repeated the analyses after expressing Wmax relative to body weight (Wmax/kg) and after estimating maximal oxygen uptake (VO_2_max) using a validated equation [33]. Sixth, we repeated the analysis of depression diagnosis, considering a composite of ICD‐10 codes F32 and F33. Seventh, we repeated the analysis of antidepressants after defining the outcome as dispensation of antidepressants on two separate occasions in contrast to a single dispensation as in the main analysis. Eighth, we repeated the analysis after excluding everyone with a history of psychiatric disorders or symptoms based on ICD‐8 and 9 codes (290‐319) as registered by the physician in charge of the medical examination during conscription. Finally, as an alternative to complete‐case analysis, we applied multiple imputation with chained equations (*K* = 20 repetitions) and performed this separately for the full cohort and the sibling cohort, using linear and multinomial logistic models for continuous (fitness, BMI and IQ) and categorical variables (parental education and income). Age at conscription, year of conscription, follow‐up time and censoring were used as complete auxiliaries, whereas body weight and height at conscription were treated as partially observed auxiliaries (imputed using a linear model).

## Results

### Baseline characteristics

The characteristics of the 1,013,885 young men in the full cohort were similar to the 410,198 siblings (Table 1). The mean age at conscription was 18.3 years (93.1% were between 15 and 19 years of age), over 80% were normal weight, one in four had parents with a high (post‐secondary) level of education and one in three had parents with a high (top category) level of annual income. Compared to conscripts in the first decile of fitness, conscripts with higher fitness had a slightly higher median birth year as well as a slightly higher mean BMI and IQ. They also had parents with a slightly higher level of education and income (Table S1).

**Table 1:** Baseline characteristics in the full cohort and the sibling cohort.

	Full cohort (*N* = 1,013,885)	Sibling cohort (*N *= 410,198)
**Birth year, median (IQR)**	1965 (1959–1970)	1965 (1959–1969)
**Age at conscription, mean (SD)**	18.3 (0.7)	18.3 (0.6)
**Body mass index, kg/m^2^, mean (SD)**	21.7 (2.8)	21.7 (2.8)
**Body mass index categories, *n* (%)**		
Underweight (<18.5 kg/m^2^)	81,745 (8.1)	33,275 (8.1)
Normal weight (18.5–24.9 kg/m^2^)	824,468 (81.3)	335,759 (81.9)
Overweight (25.0–29.9 kg/m^2^)	90,664 (8.9)	34,844 (8.5)
Obesity (>30.0 kg/m^2^)	17,008 (1.7)	6320 (1.5)
**IQ, mean (SD)**	100 (15)	100 (15)
**Cardiorespiratory fitness, Wmax, mean (SD)**	274 (52)	274 (52)
**Wmax by deciles, median (range)**		
Decile 1	202 (100–211)	203 (100–211)
Decile 2	222 (212–229)	223 (212–229)
Decile 3	236 (230–241)	236 (230–241)
Decile 4	250 (242–254)	250 (242–254)
Decile 5	262 (255–270)	262 (255–270)
Decile 6	277 (271–283)	277 (271–282)
Decile 7	291 (284–300)	291 (283–300)
Decile 8	311 (301–320)	310 (301–319)
Decile 9	332 (321–343)	330 (320–342)
Decile 10	365 (344–800)	364 (343–800)
**Parental level of education, *n* (%)**		
Compulsory school <9 years	300,122 (29.6)	125,749 (30.7)
Secondary education	453,851 (44.8)	179,448 (43.8)
Post‐secondary education <3 years	108,072 (10.7)	41,318 (10.1)
Post‐secondary education >3 years	151,840 (15.0)	63,683 (15.5)
**Parental highest income, *n* (%)**		
Category 1 (low income)	49,992 (4.9)	16,348 (4.0)
Category 2	97,735 (9.6)	35,796 (8.7)
Category 3	215,920 (21.3)	87,987 (21.5)
Category 4	307,085 (30.3)	126,230 (30.8)
Category 5 (high income)	343,153 (33.9)	143,837 (35.1)

Abbreviations: IQR = interquartile range; SD = standard deviation.

### Depression and antidepressants during follow‐up

Participants were followed until a median age (interquartile range) of 56.8 (17.7–73.5) years, during which 47,283 (4.7%) were diagnosed with depression and 237,409 (23.4%) were dispensed antidepressants (18,150 [4.4%] and 93,150 [22.7%], respectively, among siblings) (Fig. S3, Tables S2 and S3). There was a large overlap (tetrachoric correlation: 0.78) between depression diagnosis and dispensation of antidepressants, but only 18.1% of those who dispensed antidepressants were also diagnosed with depression in the patient register (Table S4). The median age at first depression diagnosis was 45.5 (39.4–51.5) years in the full cohort and 45.9 (40–51.8) among siblings. The median age at first dispensation of antidepressants was 47.4 (41.2–59.1) years in the full cohort and 47.7 (41.9–54.4) among siblings.

### Adolescent cardiorespiratory fitness and incident depression and dispensation of antidepressants

**Fig. 1:**
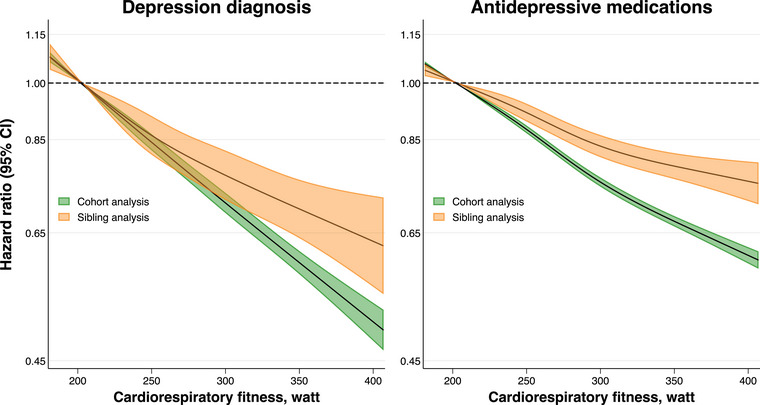
Hazard ratio for depression diagnosis and dispensation of antidepressive medications across restricted cubic splines of cardiorespiratory fitness in cohort and sibling analysis. Estimates were obtained using flexible parametric survival models, extended to a marginalized between–within model in the sibling cohort, with baseline knots placed at the 5th, 27.5th, 50th, 72.5th and 95th percentiles of the uncensored log survival times, and using age as the underlying time scale. The knots for the splines were placed at the 5th, 35th, 65th and 95th percentiles. The referent was set to the median value of the bottom decile (202 Wmax). The models were adjusted for age at conscription, year of conscription, body mass index, IQ, parental education and parental income. For graphical purposes, the x‐axis was limited to span from the 1st to the 99th percentile of the exposure distribution.

**Fig. 2:**
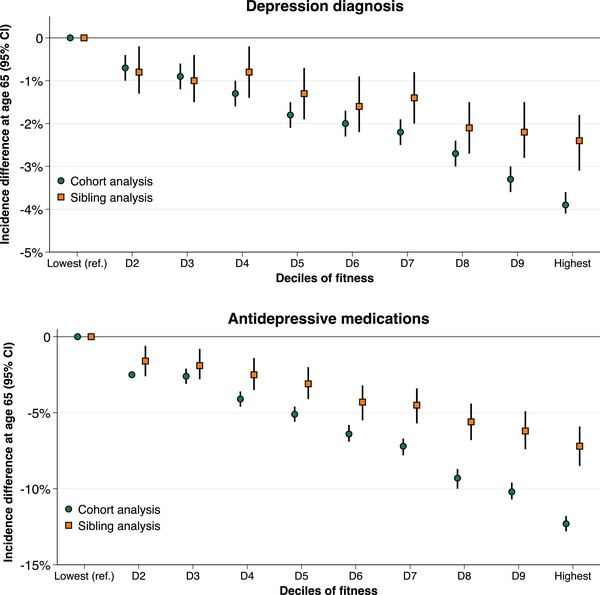
**Differences in the standardized cumulative incidence of depression diagnosis and dispensation of antidepressive medications at 65 years of age by deciles of cardiorespiratory fitness in cohort and sibling analysis**. Estimates were obtained using flexible parametric survival models, extended to a marginalized between–within model in the sibling cohort, with baseline knots placed at the 5th, 27.5th, 50th, 72.5th and 95th percentiles, and using age as the underlying time scale. The referent was the bottom decile. The models were adjusted for age at conscription, year of conscription, body mass index, IQ, parental education and parental income.

**Fig. 3:**
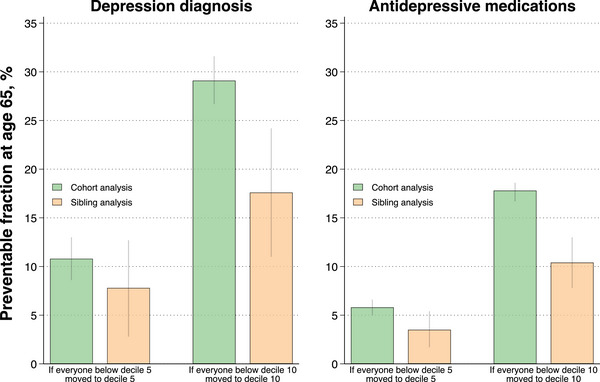
**Estimated preventable fraction of depression diagnosis and antidepressive medications at 65 years of age associated with a hypothetical moderate and extreme public health intervention in cohort and sibling analysis**. The extreme intervention entails shifting everyone to the top decile of cardiorespiratory fitness, whereas the moderate intervention involves shifting everyone from the lowest to the middle decile of fitness. Error bars indicate 95% confidence intervals.

In cohort analysis, higher fitness levels were associated with a lower risk of diagnosis of depression and dispensation of antidepressants in a linear dose–response fashion (Figs. 1 and 2, Tables S2 and S5). Compared to the first decile, the adjusted HR for depression in the 10th decile was 0.54 (95% confidence interval [CI], 0.52–0.57), and for antidepressants, 0.63 (0.62–0.65). The standardized cumulative incidence of depression by age 65 was 8.7% (8.5–8.9) in the first decile and 4.8% (4.7–5.0) in the 10th decile, with a difference of −3.9% (−4.1 to −3.6). For antidepressants, the standardized cumulative incidence by age 65% was 42.0% (41.6–42.3) in the first decile and 29.7% (29.3–30.0) in the 10th decile, with a difference of −12.3% (−12.8 to −11.8). The preventable fraction at age 65 associated with hypothetically shifting everyone from the lowest deciles to the middle decile was 10.8% (8.6–13.0) for depression and 5.8% (5.0–6.6) for antidepressants. The preventable fraction associated with hypothetically shifting everyone to the top decile was 29.1% (26.7–31.6) for depression and 17.8% (16.7–18.6) for antidepressants (Fig. 3, Table S6).

Furthermore, explorative analyses did not suggest effect modification by overweight status, with similar associations in those with overweight compared to in those without (Table S7).

### Sibling‐comparison analyses

When comparing full siblings and thus controlling for all unobserved confounders shared between them, the magnitude of the associations attenuated (Figs. 1 and 2, Table 2, Table S5). Compared to the first decile of fitness, the adjusted HR in the tenth decile was 0.67 (0.59–0.75) for depression and 0.76 (0.72–0.80) for antidepressants, with incidence differences at age 65 of −2.4% (−3.1 to −1.8) and −7.2% (−8.5 to −5.9), respectively. The preventable fraction associated with shifting everyone from the lowest deciles to the middle decile was 7.8% (2.8–12.7) for depression and 3.5% (1.7–5.4) for antidepressants, whereas hypothetically shifting everyone to the top decile was associated with a preventable fraction of 17.6% (11.0–24.2) for depression and 10.4% (7.8–13.0) for antidepressants (Fig. 3, Table S6).

**Table 2:** Hazard ratios and standardized cumulative incidences of depression diagnosis and dispensation of antidepressive medications by deciles of cardiorespiratory fitness in cohort and sibling analysis.

Depression diagnosis								
	Cohort analysis (*N* = 1,013,885)				Sibling analysis (*N* = 410,198)			
Deciles of fitness	Cases/N	HR (95% CI)	Standardized incidence at age 65, % (95% CI)	Incidence difference, % (95% CI)	Cases/N	HR (95% CI)	Standardized incidence at age 65, % (95% CI)	Incidence difference, % (95% CI)
D1	6905/113,887	Ref.	8.7 (8.5, 8.9)	Ref.	2675/45,454	Ref.	7.5 (7.1, 7.9)	Ref.
D2	5392/100,857	0.91 (0.88, 0.95)	8.0 (7.7, 8.2)	−0.7 (−1.0, −0.4)	2114/41,317	0.89 (0.82, 0.97)	6.7 (6.3, 7.2)	−0.8 (−1.3, −0.2)
D3	5094/96,126	0.89 (0.86, 0.93)	7.8 (7.6, 8.0)	−0.9 (−1.2, −0.6)	1975/39,087	0.87 (0.80, 0.94)	6.6 (6.2, 7.0)	−1.0 (−1.5, −0.4)
D4	4985/100,065	0.84 (0.81, 0.88)	7.4 (7.2, 7.6)	−1.3 (−1.6, −1.0)	1971/41,269	0.89 (0.81, 0.97)	6.7 (6.3, 7.1)	−0.8 (−1.4, −0.2)
D5	5272/110,985	0.79 (0.76, 0.82)	6.9 (6.7, 7.1)	−1.8 (−2.1, −1.5)	2016/45,169	0.82 (0.75, 0.90)	6.2 (5.9, 6.6)	−1.3 (−1.9, −0.7)
D6	4019/88,231	0.76 (0.73, 0.79)	6.7 (6.5, 6.9)	−2.0 (−2.3, −1.7)	1552/36,308	0.79 (0.71, 0.86)	6.0 (5.6, 6.4)	−1.6 (−2.2, −0.9)
D7	4672/103,963	0.74 (0.71, 0.77)	6.5 (6.3, 6.7)	−2.2 (−2.5, −1.9)	1724/41,950	0.81 (0.73, 0.89)	6.1 (5.7, 6.5)	−1.4 (−2.0, −0.8)
D8	4164/102,896	0.68 (0.65, 0.71)	6.0 (5.8, 6.2)	−2.7 (−3.0, −2.4)	1556/41,878	0.71 (0.64, 0.78)	5.4 (5.0, 5.7)	−2.1 (−2.7, −1.5)
D9	3540/96,282	0.61 (0.59, 0.64)	5.4 (5.3, 5.6)	−3.3 (−3.6, −3.0)	1307/37,917	0.70 (0.63, 0.78)	5.3 (4.9, 5.7)	−2.2 (−2.8, −1.5)
D10	3240/100,593	0.54 (0.52, 0.57)	4.8 (4.7, 5.0)	−3.9 (−4.1, −3.6)	1260/39,849	0.67 (0.59, 0.75)	5.1 (4.7, 5.5)	−2.4 (−3.1, −1.8)

Dispensation of antidepressive medications								
D1	32,125/113,887	Ref.	42.0 (41.6, 42.3)	Ref.	12,512/45,454	Ref.	37.4 (36.7, 38.2)	Ref.
D2	26,175/100,857	0.92 (0.90, 0.93)	39.5 (39.1, 39.9)	−2.5 (−2.9, −2.0)	10,516/41,317	0.94 (0.91, 0.98)	35.8 (35.0, 36.6)	−1.6 (−2.6, −0.6)
D3	24,919/96,126	0.92 (0.90, 0.93)	39.4 (39.0, 39.8)	−2.6 (−3.1, −2.1)	9936/39,087	0.93 (0.90, 0.97)	35.5 (34.7, 36.3)	−1.9 (−2.9, −0.8)
D4	24,668/100,065	0.87 (0.85, 0.88)	37.8 (37.5, 38.2)	−4.1 (−4.6, −3.6)	9786/41,269	0.91 (0.88, 0.95)	34.9 (34.2, 35.7)	−2.5 (−3.5, −1.4)
D5	26,702/110,985	0.84 (0.82, 0.85)	36.9 (36.5, 37.2)	−5.1 (−5.6, −4.6)	10,556/45,169	0.89 (0.86, 0.93)	34.3 (33.6, 35.0)	−3.1 (−4.1, −2.0)
D6	20,319/88,231	0.80 (0.78, 0.81)	35.6 (35.2, 36.0)	−6.4 (−6.9, −5.8)	8081/36,308	0.85 (0.82, 0.89)	33.1 (32.2, 33.8)	−4.3 (−5.5, −3.2)
D7	23,332/103,963	0.77 (0.76, 0.79)	34.7 (34.4, 35.1)	−7.2 (−7.8, −6.7)	8969/41,950	0.85 (0.81, 0.88)	32.9 (32.1, 33.6)	−4.5 (−5.7, −3.4)
D8	21,379/102,896	0.72 (0.70, 0.73)	32.7 (32.4, 33.1)	−9.3 (−10.0, −8.7)	8409/41,878	0.81 (0.78, 0.85)	31.8 (31.1, 32.6)	−5.6 (−6.8, −4.4)
D9	19,256/96,282	0.69 (0.68, 0.70)	31.8 (31.4, 32.2)	−10.2 (−10.7, −9.6)	7231/27,243	0.79 (0.76, 0.83)	31.2 (30.4, 32.1)	−6.2 (−7.4, −4.9)
D10	18,534/100 593	0.63 (0.62, 0.65)	29.7 (29.3, 30.0)	−12.3 (−12.8, −11.8)	7154/39,849	0.76 (0.72, 0.80)	30.2 (29.3, 31.1)	−7.2 (−8.5, −5.9)

*Note*: The models were adjusted for age at conscription, year of conscription, body mass index, IQ, parental education and parental income.

Abbreviations: CI, confidence interval; D, decile; HR, hazard ratio.

### Sensitivity analyses

Replication of the standard analysis in the sibling cohort produced similar estimates as observed in the cohort analysis (Table S8). Restricting the analysis to those who conscribed in the year 1985 and later produced similar estimates for antidepressants but stronger estimates for the outcome of depression diagnosis, although differences between cohort and sibling analysis remained (Table S9). The findings were similar when the year of conscription was modelled using restricted cubic splines and when it was conditioned on using stratified Cox regression (Tables S10 and S11). Allowing the effect of fitness to vary over time did not yield materially different estimates compared to the main analysis (Table S12). The results were similar when using the exposures Wmax/kg and estimated VO_2_max compared to Wmax (Table S13). The results were also similar for the composite outcome considering both F32 and F33 (Table S14), when requiring two dispensations of antidepressants (Table S15), when excluding everyone with pre‐existing psychiatric disorders at baseline (Table S16), and when using multiple imputation (Table S17).

## Discussion

In this nationwide cohort study encompassing more than 1 million men, high levels of adolescent cardiorespiratory fitness were associated with a lower risk of adulthood depression and dispensation of antidepressants in a linear dose–response fashion. However, the associations attenuated in sibling‐comparison analyses, suggesting that the findings may be influenced by familial confounding.

Our findings extend upon previous studies which have found a link between midlife fitness (typically around 40–50 years of age) and subsequent depression and antidepressant dispensation [7, 8, 10, 11, 12, 13]. The role of specifically adolescent fitness has remained largely underexplored, although Åberg et al. reported an excess risk of adulthood depression among Swedish male conscripts with low fitness levels [9]. In our study, we examined the dose–response association with even longer follow‐up and greater granularity, observing lower risks already in the second decile of fitness, and the association did not seem to plateau at high levels of fitness. Our findings, regardless if proven to be causal, suggest that monitoring fitness from a young age may offer important insights into their long‐term risk of mental health problems, rendering support to ongoing surveillance programs [34, 35].

Notwithstanding this, an important question for public health and prevention is whether findings from standard observational analyses also adequately reflect a causal relationship, which would support targeted interventions, or if there are alternative explanations to the association. We found that although higher adolescent fitness was associated with lower risks of future depression and antidepressants even after controlling for unobserved confounders shared between full siblings, the magnitude of the relationships attenuated. In order to facilitate clinical interpretation of the attenuation magnitude, we not only estimated HRs [36], but we also estimated absolute cumulative risks and preventable fractions both before and after controlling for unobserved confounders shared between full siblings. Interestingly, the standardized incidence differences comparing the most fit to the least fit were notably reduced from cohort to sibling analysis: from −3.9% to −2.4% for depression and from −12.3% to −7.2% for antidepressants. The potential public health implications of these changes are further reflected by the attenuation in the fraction of preventable cases (i.e., from 29.1% to 17.6% for depression and from 17.8% to 10.4% for antidepressants). This indicates that a sizeable proportion of the cases assumed to be preventable from improving fitness when performing standard cohort analysis may be due to familial confounding. Although the sibling‐comparison analysis cannot pinpoint the definitive sources of familial confounders and their relative contribution, it may include a combination of behavioural (e.g., clustering of lifestyle habits), environmental (e.g., socioeconomic factors and upbringing) and genetic influences. It is crucial to emphasize that there are strong assumptions underlying estimations of preventable fractions; hence, we caution against interpreting the estimates as definitive. Nevertheless, the comparison of the preventable fractions between cohort and sibling analysis may be viewed as a relevant measure of potential public health impact, where the contrast sheds light on the potential overestimation of the effect.

To our knowledge, no previous study has employed methods to control for familial confounding in studies on cardiorespiratory fitness and depression. Two studies did, however, incorporate twin comparators in studies on physical activity in relation to depressive and anxiety symptoms and use of antidepressants in predominantly middle‐aged individuals, reporting conflicting findings [37, 38]. Moreover, using a Mendelian randomization framework, Choi et al. found that genetic variants for device‐measured physical activity, but not self‐reported activity, were associated with a lower risk of depression [39]. Besides the difference in the exposures (physical activity vs. fitness), those estimates were unfortunately not contrasted to those derived using standard cohort analysis. Thus, our study adds new evidence by comparing standard observational estimates with those derived using sibling‐comparisons and, as such, demonstrating the importance of triangulation of evidence across methods to better understand the link between adolescent cardiorespiratory fitness and future risk of depressive disorders. Given the scarcity of evidence, further causal analyses are still warranted, including in studies incorporating longitudinal measures of fitness.

Another interesting finding was that the associations appeared similar among individuals with overweight compared to those without overweight. Although these analyses should be carefully interpreted as they were exploratory and limited in statistical power due to the small number of individuals with overweight or obesity, they may be particularly valuable for future meta‐analyses because those conducted to date have not been able to examine this question [7, 8]. It would also be valuable with similar analyses based on contemporary cohorts with higher overweight/obesity prevalence, as these might yield different results.

### Strengths and limitations

Strengths of our study include the use and linkage of high‐quality nationwide registers, allowing us to follow a large cohort of young men with standardized measures of fitness for several decades with virtually zero loss to follow‐up. This provided high statistical power and enabled detailed analyses, including for sibling‐comparisons. The latter also allowed for triangulation of the evidence via control for some unobserved familial confounders, in contrast to previous studies which typically relied on conventional analytical approaches.

There are also limitations. Due to historical conscription practices in Sweden, only men were included. Because the prevalence of depressive disorders is higher among women [1], as well as evidence suggesting that sex might modify the association between fitness and depression [12, 13], and that the genetic influence in depression may be higher in women [40], further studies incorporating sibling‐comparisons and other methods to strengthen causal inference in women are warranted. Regarding the ascertainment of depression diagnosis, we relied on specialized care data using the National Patient Register, which is likely to lead to an under‐ascertainment of cases given that most (e.g., 81% in one study) patients are treated exclusively in primary care [41]. This could introduce misclassification bias, where if uncaptured cases were evenly distributed across fitness levels, the association might be biased towards the null. In contrast, if uncaptured cases differed with respect to health status, including fitness levels and/or severity of depression, the association might be biased away from the null. To target this limitation, we incorporated dispensed antidepressants as an outcome. Despite not being a perfect substitute, this outcome helps to triangulate the findings, as it has been estimated that between 58% and 76% of patients with depression that are treated exclusively in primary care are also using antidepressants [41, 42]. As such, we were likely to counteract some of the under‐ascertainment by also examining this outcome. Nonetheless, some under‐ascertainment likely remains, as individuals who never attended specialized outpatient care but still received cognitive behavioural therapy (first‐line treatment) and never dispensed antidepressants are not covered by our data. Regardless, any under‐ascertainment is unlikely to explain the changes in estimates from cohort to sibling analysis. Additionally, because ICD‐10 was not officially implemented until 1997 and the Prescribed Drug Register was not launched until 2005, the age of first diagnosis and dispensation of medication was likely overestimated; the data are somewhat left‐truncated to 1997 due to the coverage of the registries and ICD codes. Again, this is unlikely to exert an influence on the differences observed between estimates in cohort compared to sibling analysis, as was also confirmed in a sensitivity analysis restricted to later cohorts. Nevertheless, the incidence might be underestimated if individuals who were diagnosed/dispensed medications prior to 1997/2005 later went into remission and none of their subsequent records noted such a history. Whether this bias would be differential or nondifferential can only be speculated. Another limitation regarding diagnoses is that we relied on registry data and were not able to clinically validate the cases. We also lacked information on certain confounders, such as somatic health conditions, loneliness, stress, alcohol, smoking and other lifestyle factors such as physical activity. Further, although we compared full siblings and thus controlled for all factors that they share, residual confounding remains possible, including from non‐shared behaviours, environmental factors and genetics. Future studies based on monozygotic twins with in‐depth phenotyping would therefore be valuable. Finally, although the sibling‐comparison design enables control for unobserved shared factors, it hinges on assumptions including (but not limited to) the absence of non‐shared confounding and no measurement error, which, if present and not accounted for, can amplify bias, which could then explain the attenuated estimates in sibling analysis [43].

## Conclusion

High levels of adolescent cardiorespiratory fitness are associated with a lower risk of future depression diagnosis and dispensation of antidepressants. However, the associations become weaker when comparing full siblings and thereby accounting for all factors shared between them, suggesting that unobserved familial confounding may overestimate the association. These findings highlight the challenges of obtaining valid estimates of the causal relationship between cardiorespiratory fitness and depression and underscore the need for further causal analyses to establish more robust conclusions.

## Author contributions

**Marcel Ballin**: Conceptualization; writing—original draft; writing—review and editing; methodology; formal analysis; visualization; investigation; validation; project administration. **Örjan Ekblom**: Writing—review and editing; methodology; investigation. **Anna Nordström**: Writing—review and editing; methodology; investigation. **Viktor H. Ahlqvist**: Writing—review and editing; data curation; methodology; investigation; project administration. **Peter Nordström**: Conceptualization; data curation; supervision; validation; project administration; writing—review and editing; methodology; investigation.

## Conflict of interest statement

M.B. is employed at the Swedish Medical Products Agency, SE‐751 03 Uppsala, Sweden. The views expressed in this article do not necessarily represent the views of this Government agency. All other authors declare no conflicts of interest.

## Funding information

The author(s) received no specific funding for this work.

## Ethics statement

The study was approved by the Regional Ethical Review Board in Umeå and later by the Swedish Ethical Review Authority (no. 2010‐113‐31M), which waived the need for informed consent.

## Supporting information




**Figure S1**: Participant flow chart. Created with BioRender.com.
**Figure S2**: Directed acyclic graph for the association between adolescent cardiorespiratory fitness and risk of depression in late adulthood, where observed (white) and unobserved (grey) confounders are illustrated. Created in BioRender. Ahlqvist, V. (2025) https://BioRender.com/sitsvs1.
**Figure S3**: Frequency of depression (top) and antidepressants (bottom) during follow‐up by calendar year.
**Table S1**: Baseline characteristics by deciles of cardiorespiratory fitness in the full cohort and in the sibling cohort.
**Table S2**: Numbers censored due to death, emigration and end of follow‐up.
**Table S3**: Subtypes of depression diagnoses among individuals with a depression outcome during follow‐up.
**Table S4**: Overlap between individuals with a depression diagnosis in the National Patient Register and individuals with a dispensation of antidepressants in the Prescribed Drug Register.
**Table S5**: Unadjusted hazard ratios for depression diagnosis and dispensation of antidepressive medications by deciles of cardiorespiratory fitness in the full cohort.
**Table S6**: Estimated preventable fraction of depression diagnosis and dispensation of antidepressive medications at 65 years of age associated with a moderate (shifting those below deciles 5 to 5) or an extreme hypothetical intervention (shifting everyone to decile 10) in cohort and sibling analysis.
**Table S7**: Hazard ratios for depression diagnosis and dispensation of antidepressive medications by deciles of cardiorespiratory fitness in cohort and sibling analysis, with and without allowing for effect modification by overweight, and in strata of overweight status.
**Table S8**: Hazard ratios for depression diagnosis and dispensation of antidepressive medications by deciles of cardiorespiratory fitness in cohort analysis (as reported in the main article), in the sibling cohort using standard analysis, and using sibling analysis (as reported in the main article).
**Table S9**: Hazard ratios for depression diagnosis and dispensation of antidepressive medications by deciles of cardiorespiratory fitness in cohort and sibling analysis, restricted to those who conscribed in the year 1985 or later.
**Table S10**: Hazard ratios for depression diagnosis and dispensation of antidepressive medications by deciles of cardiorespiratory fitness in cohort and sibling analysis, modelling the covariate year of conscription using restricted cubic splines versus as a categorical variable as in the main analysis.
**Table S11**: Hazard ratios for depression diagnosis and dispensation of antidepressive medications by deciles of cardiorespiratory fitness in cohort analysis, including regression adjustment for year of conscription as in the main analysis versus using stratified Cox regression conditioned on year of conscription.
**Table S12**: Standardized cumulative incidences of depression diagnosis and dispensation of antidepressive medications at 65 years of age by deciles of cardiorespiratory fitness in cohort and sibling analysis, allowing the effect of fitness to vary across follow‐up time^a^.
**Table S13**: Hazard ratios for depression diagnosis and dispensation of antidepressive medications by deciles of cardiorespiratory fitness expressed as Wmax (as reported in the main article), Wmax/kg or estimated VO_2_max in cohort and sibling analysis.
**Table S14**: Hazard ratios for depression diagnosis (F32 and F33) by deciles of cardiorespiratory fitness in cohort and sibling analysis.
**Table S15**: Hazard ratios for a first versus second dispensation of antidepressive medications by deciles of cardiorespiratory fitness in cohort and sibling analysis.
**Table S16**: Hazard ratios for depression diagnosis and dispensation of antidepressive medications by deciles of cardiorespiratory fitness in cohort and sibling analysis, excluding everyone with pre‐existing psychiatric disorders or symptoms at baseline.
**Table S17**: Hazard ratios for depression diagnosis and dispensation of antidepressive medications by deciles of cardiorespiratory fitness in cohort and sibling analysis using complete‐case analysis versus using multiple imputation.

## Data Availability

The data in this study are not available to the public and will not be shared according to regulations under Swedish law. Researchers interested in obtaining the data may seek ethical approvals and inquire through Statistics Sweden. For further advice, see https://www.scb.se/en/services/ordering‐data‐and‐statistics/. Analytical code underlying the results is available from the corresponding author upon request.
