# Supplementary material for: Overstated association between adolescent physical fitness and adulthood depression risk due to familial factors
Source: J Intern Med. 2025 Jul 9;298(3):200–13. doi: 10.1111/joim.20109 (PMC12374760; doi:10.1111/joim.20109)
Supplement: Supplementary file 1 — Figure S1: Participant flow chart. Created with BioRender.com. Figure S2: Directed acyclic graph for the association between adolescent cardiorespiratory fitness and risk of depression in late adulthood, where observed (white) and unobserved (grey) confounders are illustrated. Created in BioRender. Ahlqvist, V. (2025) https://BioRender.com/sitsvs1. Figure S3: Frequency of depression (top) and antidepressants (bottom) during follow‐up by calendar year. Table S1: Baseline characteristics by deciles of cardiorespiratory fitness in the full cohort and in the sibling cohort. Table S2: Numbers censored due to death, emigration and end of follow‐up. Table S3: Subtypes of depression diagnoses among individuals with a depression outcome during follow‐up. Table S4: Overlap between individuals with a depression diagnosis in the National Patient Register and individuals with a dispensation of antidepressants in the Prescribed Drug Register. Table S5: Unadjusted hazard ratios for depression diagnosis and dispensation of antidepressive medications by deciles of cardiorespiratory fitness in the full cohort. Table S6: Estimated preventable fraction of depression diagnosis and dispensation of antidepressive medications at 65 years of age associated with a moderate (shifting those below deciles 5 to 5) or an extreme hypothetical intervention (shifting everyone to decile 10) in cohort and sibling analysis. Table S7: Hazard ratios for depression diagnosis and dispensation of antidepressive medications by deciles of cardiorespiratory fitness in cohort and sibling analysis, with and without allowing for effect modification by overweight, and in strata of overweight status. Table S8: Hazard ratios for depression diagnosis and dispensation of antidepressive medications by deciles of cardiorespiratory fitness in cohort analysis (as reported in the main article), in the sibling cohort using standard analysis, and using sibling analysis (as reported in the main article). Table S9: Hazard rati [file JOIM-298-200-s001.docx]

Supplemental material

# Overstated association between adolescent physical fitness and adulthood depression risk due to familial factors

Marcel Ballin, PhD (ORCID: 0000-0002-9638-7208)^1^, Örjan Ekblom, PhD (ORCID: 0000-0001-6058-4982)^2^, Anna Nordström, MD, PhD (ORCID: 0000-0003-3534-456X)^3,4^, Viktor H. Ahlqvist, PhD (ORCID: 0000-0003-1383-3194)^1,5,6^, Peter Nordström, MD, PhD (ORCID: 0000-0003-2924-508X)^1^

^1^Department of Public Health and Caring Sciences, Clinical Geriatrics, Uppsala University, Uppsala, Sweden.

^2^Department of Physical Activity and Health, The Swedish School of Sport and Health Sciences, Stockholm, Sweden.

^3^Department of Medical Sciences, Rehabilitation Medicine, Uppsala University, Uppsala, Sweden.

^4^School of Sport Sciences, UiT The Arctic University of Norway, Tromsø, Norway.

^5^Department of Biomedicine, Aarhus University, Aarhus, Denmark.

^6^Institute of Environmental Medicine, Karolinska Institutet, Stockholm, Sweden.

**
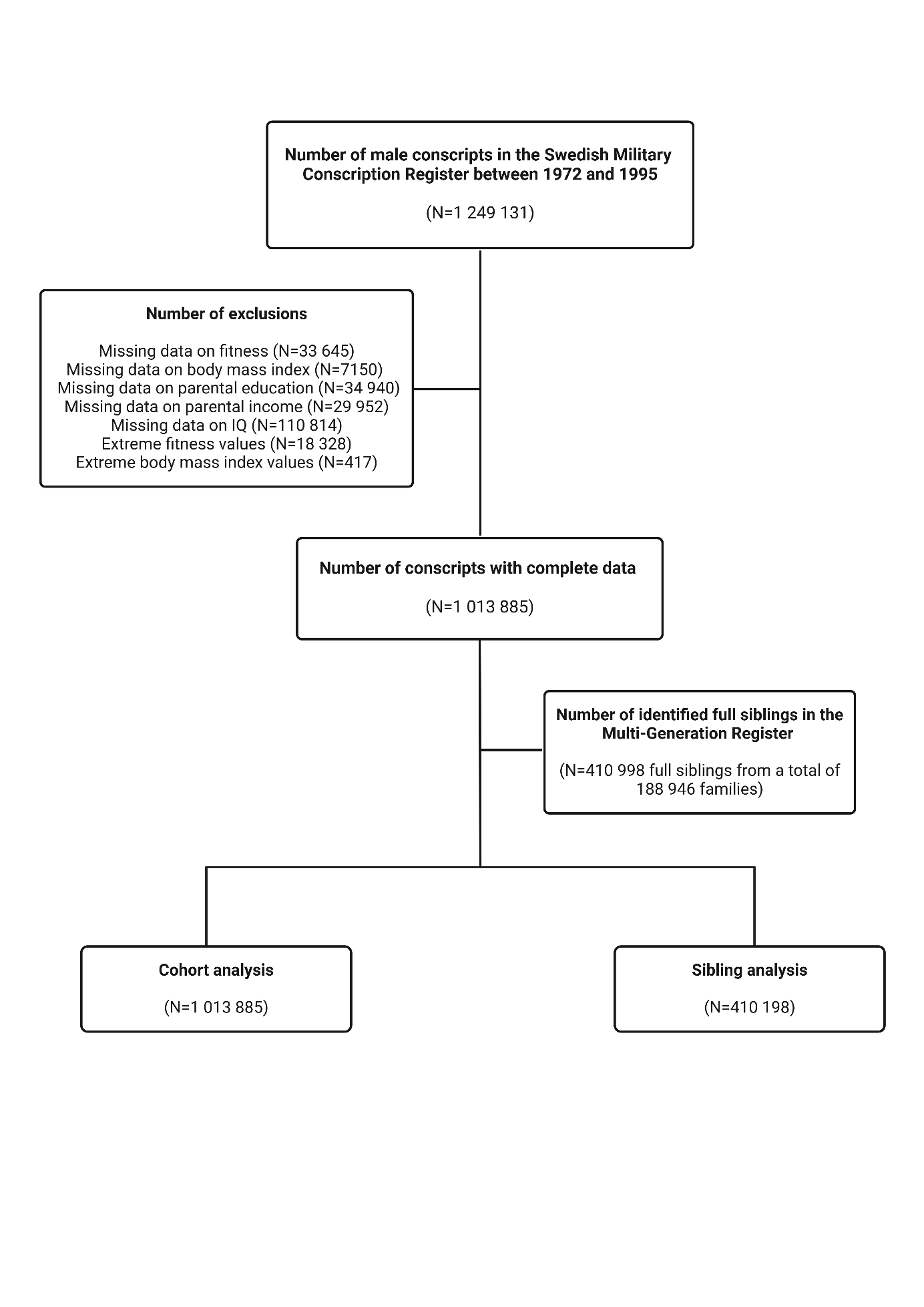
**

**Supplemental figure 1. Participant flow chart.** Created with BioRender.com


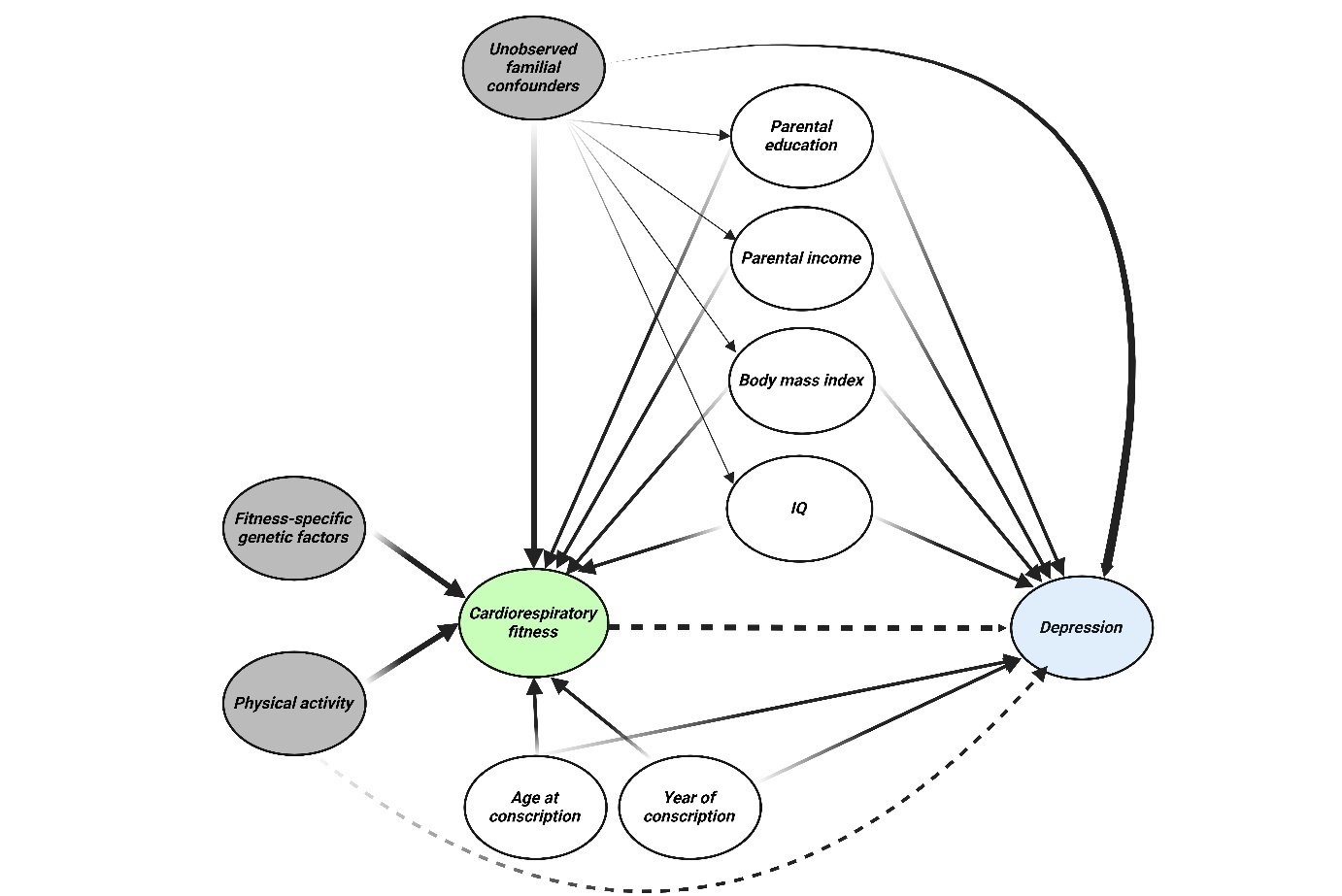


Supplemental figure 2. **Directed acyclic graph for the association between adolescent cardiorespiratory fitness and risk of depression in late adulthood, where observed (white) and unobserved (gray) confounders are illustrated**. Created in BioRender. Ahlqvist, V. (2025) https://BioRender.com/sitsvs1


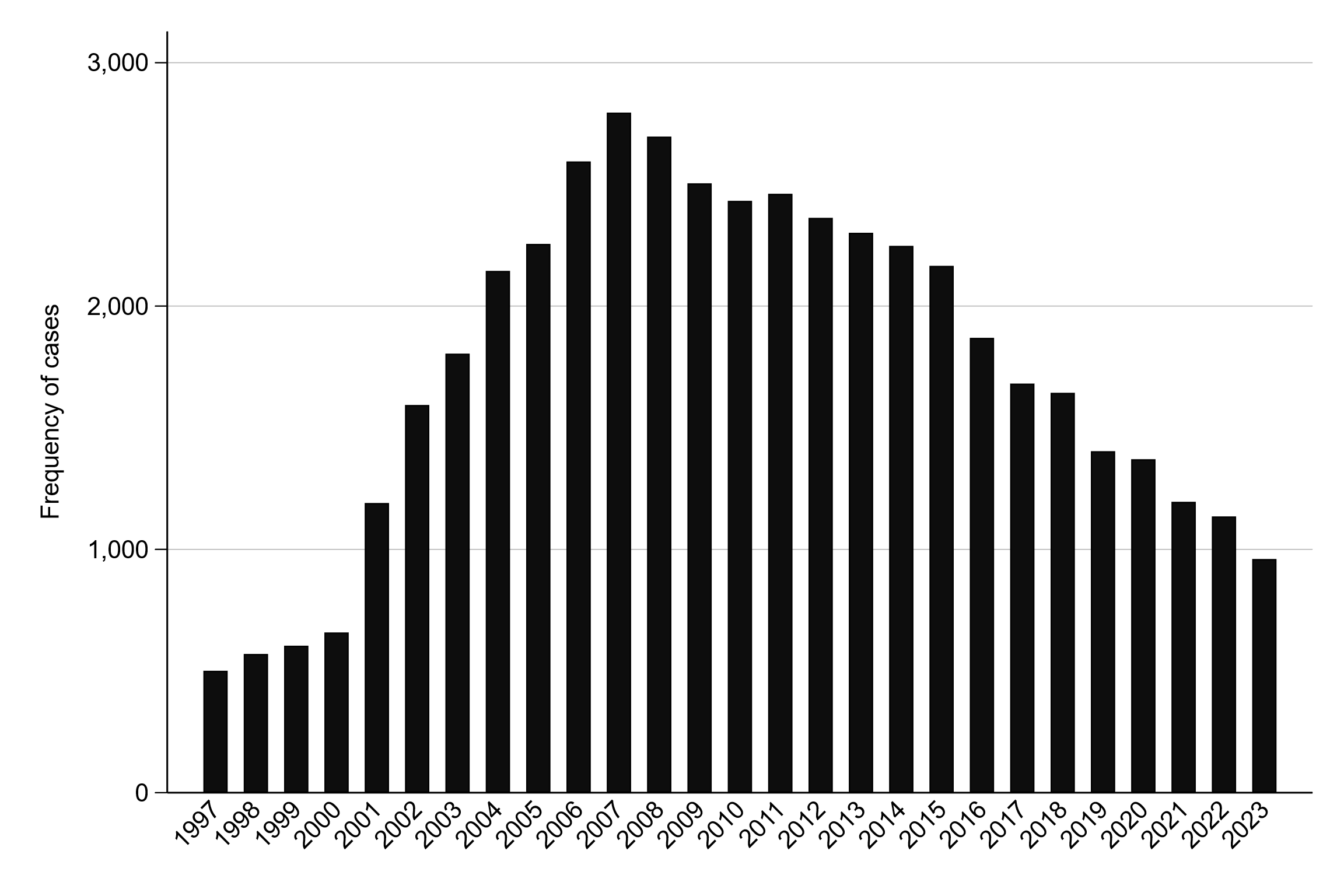


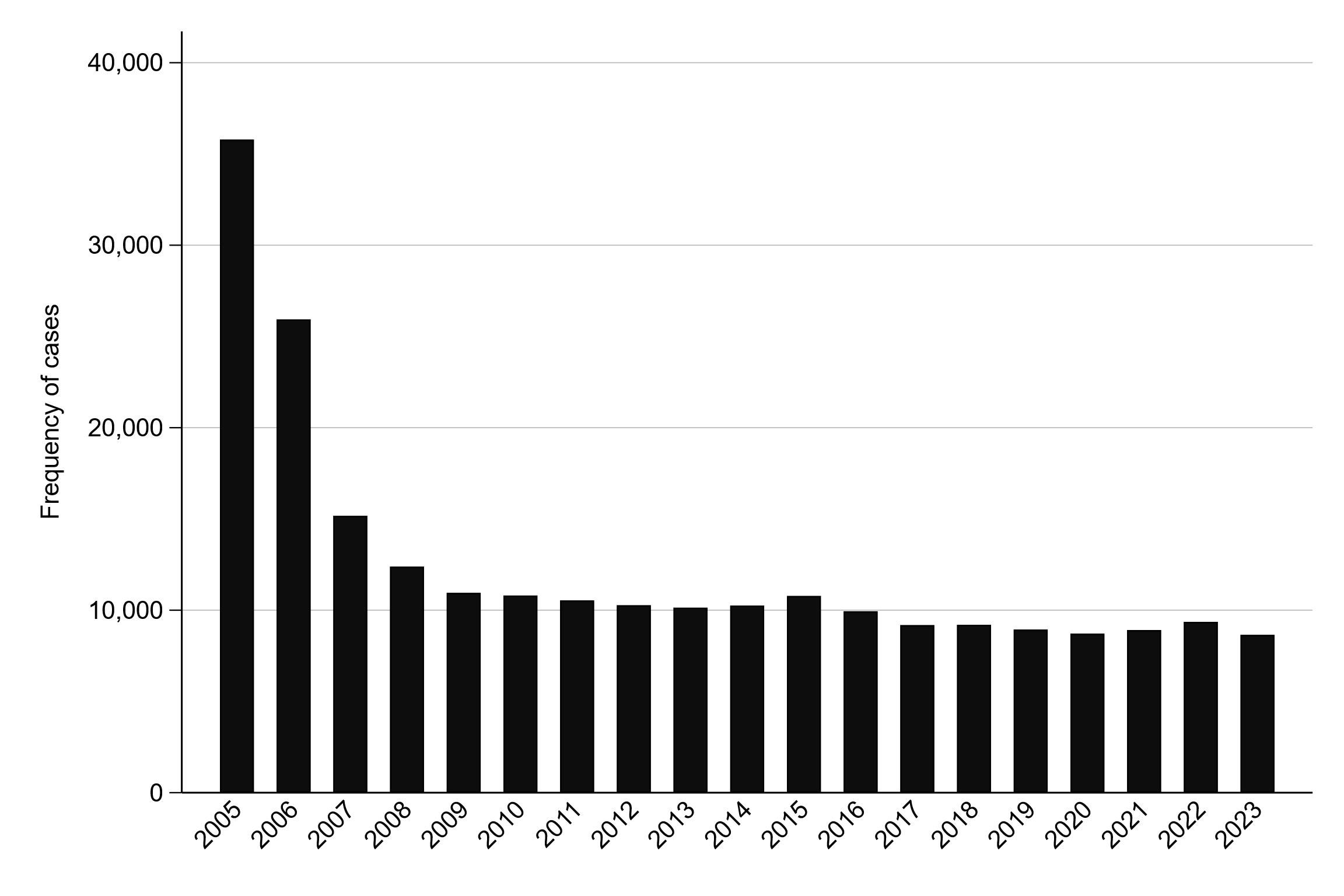


Supplemental figure 3. **Frequency of depression (top) and antidepressants (bottom) during follow-up by calendar year.**

| **Supplemental table 1. Baseline characteristics by deciles of cardiorespiratory fitness in the full cohort and in the sibling cohort.** | | | | | | | | | | |
| --- | --- | --- | --- | --- | --- | --- | --- | --- | --- | --- |
|  | **Full cohort** | | | | | | | | | |
| **Variables** | **Decile 1 (N=113 887)** | **Decile 2**  **(N=100 857)** | **Decile 3**  **(N=96 126)** | **Decile 4**  **(N=100 065)** | **Decile 5**  **(N=110 985)** | **Decile 6**  **N=88 231)** | **Decile 7**  **(N=103 963)** | **Decile 8**  **(N=102 896)** | **Decile 9**  **(N=96 282)** | **Decile 10**  **(N=100 593)** |
| **Birth year, median (IQR)** | 1959 (1950-1976) | 1959 (1950-1976) | 1962 (1950-1976) | 1962 (1950-1977) | 1964 (1950-1977) | 1965 (1951-1977) | 1968 (1951-1977) | 1968 (1951-1977) | 1970 (1951-1977) | 1970 (1952-1977) |
| **Age at conscription, mean (SD)** | 18.4 (0.9) | 18.4 (0.8) | 18.3 (0.8) | 18.3 (0.7) | 18.3 (0.7) | 18.3 (0.6) | 18.3 (0.6) | 18.3 (0.6) | 18.3 (0.5) | 18.3 (0.5) |
| **IQ, mean (SD)** | 95.4 (15.6) | 97.8 (15.2) | 98.4 (15.1) | 99.5 (14.9) | 99.8 (14.9) | 100.7 (14.6) | 101.3 (14.5) | 102.6 (14.2) | 103.3 (13.7) | 104.5 (13.3) |
| **Body mass index, kg/m^2^, mean (SD)** | 20.2 (2.8) | 21.0 (2.7) | 21.4 (2.9) | 21.6 (2.8) | 21.8 (2.8) | 21.9 (2.7) | 22.1 (2.8) | 22.3 (2.6) | 22.3 (2.5) | 23.0 (2.6) |
| **Body mass index categories, n (%)** |  |  |  |  |  |  |  |  |  |  |
| Underweight (<18.5 kg/m^2^) | 28 940 (25.4) | 13 490 (13.4) | 9739 (10.1) | 7802 (7.8) | 7464 (6.7) | 4605 (5.2) | 4388 (4.2) | 2604 (2.5) | 2067 (2.2) | 646 (0.6) |
| Normal weight (18.5-24.9 kg/m^2^) | 78 788 (69.2) | 80 030 (79.4) | 77 270 (80.4) | 82 466 (82.4) | 91 688 (82.6) | 73 900 (83.8) | 86 937 (83.6) | 87 210 (84.8) | 83 007 (86.2) | 83 172 (82.7) |
| Overweight (25.0-29.9 kg/m^2^) | 4814 (4.2) | 6046 (6.0) | 7502 (7.8) | 8107 (8.1) | 9844 (8.9) | 8213 (9.3) | 10 538 (10.1) | 11 320 (11.0) | 9718 (10.1) | 14 562 (14.5) |
| Obesity (>30.0 kg/m^2^) | 1345 (1.2) | 1291 (1.3) | 1615 (1.7) | 1690 (1.7) | 1989 (1.8) | 1513 (1.7) | 2100 (2.0) | 1762 (1.7) | 1490 (1.6) | 2213 (2.2) |
| **Parental level of education, n (%)** |  |  |  |  |  |  |  |  |  |  |
| Compulsory school <9 years | 49 379 (43.4) | 41 529 (41.2) | 36 126 (37.6) | 34 806 (34.8) | 33 929 (30.6) | 25 090 (28.4) | 24 932 (24.0) | 22 524 (21.9) | 16 903 (17.6) | 14 904 (14.8) |
| Secondary education | 47 694 (41.9) | 42 669 (42.3) | 41 827 (43.5) | 44 282 (44.3) | 50 781 (45.8) | 40 414 (45.8) | 48 885 (47.0) | 47 487 (46.2) | 44 776 (46.5) | 45 036 (44.8) |
| Post-secondary education <3 years | 7737 (6.8) | 7465 (7.4) | 7974 (8.3) | 9119 (9.1) | 11 370 (10.2) | 9410 (10.7) | 12 563 (12.1) | 13 252 (12.9) | 13 802 (14.3) | 15 380 (15.3) |
| Post-secondary education >3 years | 9077 (8.0) | 9194 (9.1) | 10 199 (10.6) | 11 858 (11.9) | 14 905 (13.4) | 13 317 (15.1) | 17 583 (16.9) | 19 633 (19.1) | 20 801 (21.6) | 25 273 (25.1) |
| **Parental highest income, n (%)** |  |  |  |  |  |  |  |  |  |  |
| Category 1 (low income) | 7879 (6.9) | 6522 (6.5) | 5697 (5.9) | 5645 (5.6) | 5636 (5.1) | 4295 (4.8) | 4516 (4.3) | 3897 (3.8) | 3137 (3.3) | 2768 (2.8) |
| Category 2 | 13 690 (12.0) | 11 358 (11.3) | 10 445 (10.9) | 10 267 (10.3) | 11 325 (10.2) | 8231 (9.3) | 9443 (9.1) | 8570 (8.3) | 7647 (7.9) | 6759 (6.7) |
| Category 3 | 28 519 (25.0) | 24 167 (24.0) | 22 139 (23.0) | 22 436 (22.4) | 24 161 (21.8) | 18 498 (21.0) | 21 338 (20.5) | 19 739 (19.2) | 17 866 (18.6) | 17 057 (17.0) |
| Category 4 | 34 908 (30.7) | 30 443 (30.2) | 29 576 (30.8) | 30 619 (30.6) | 33 749 (30.4) | 26 847 (30.4) | 31 532 (30.3) | 30 630 (29.8) | 28 985 (30.1) | 29 796 (29.6) |
| Category 5 (high income) | 28 891 (25.4) | 28 367 (28.1) | 28 269 (29.4) | 31 098 (31.1) | 36 114 (32.5) | 30 360 (34.4) | 37 134 (35.7) | 40 060 (38.9) | 38 647 (40.1) | 44 213 (44.0) |
|  | **Sibling cohort** | | | | | | | | | |
|  | **Decile 1**  **(N=45 454)** | **Decile 2**  **(N=41 317)** | **Decile 3**  **(N=39 087)** | **Decile 4**  **(N=41 269)** | **Decile 5**  **(N=45 169)** | **Decile 6**  **(N=36 308)** | **Decile 7**  **(N=41 950)** | **Decile 8**  **(N=41 878)** | **Decile 9**  **(N=37 917)** | **Decile 10**  **(N=39 849)** |
| **Birth year, median (IQR)** | 1960 (1950-1976) | 1961 (1950-1976) | 1962 (1950-1976) | 1962 (1950-1976) | 1964 (1951-1977) | 1965 (1951-1976) | 1967 (1951-1977) | 1967 (1952-1977) | 1969 (1952-1977) | 1970 (1953-1977) |
| **Age at conscription, mean (SD)** | 18.4 (0.9) | 18.3 (0.8) | 18.3 (0.8) | 18.3 (0.7) | 18.3 (0.7) | 18.3 (0.6) | 18.3 (0.6) | 18.2 (0.5) | 18.3 (0.5) | 18.3 (0.5) |
| **IQ, mean (SD)** | 94.6 (15.6) | 97.1 (15.3) | 97.8 (15.3) | 99.0 (15.1) | 99.6 (15.0) | 100.6 (14.7) | 101.2 (14.6) | 102.6 (14.3) | 103.2 (13.8) | 104.7 (13.4) |
| **Body mass index, kg/m^2^, mean (SD)** | 20.2 (2.7) | 20.9 (2.7) | 21.4 (2.8) | 21.5 (2.8) | 21.7 (2.8) | 21.8 (2.7) | 22.0 (2.8) | 22.3 (2.6) | 22.2 (2.5) | 22.9 (2.6) |
| **Body mass index categories, n (%)** |  |  |  |  |  |  |  |  |  |  |
| Underweight (<18.5 kg/m^2^) | 11 711 (25.8) | 5646 (13.7) | 3941 (10.1) | 3167 (7.7) | 3009 (6.7) | 1868 (5.1) | 1742 (4.2) | 1078 (2.6) | 840 (2.2) | 273 (0.7) |
| Normal weight (18.5-24.9 kg/m^2^) | 31 449 (69.2) | 32 801 (79.4) | 31 550 (80.7) | 34 267 (83.0) | 37 575 (83.2) | 30 678 (84.5) | 35 394 (84.4) | 35 772 (85.4) | 32 875 (86.7) | 33 398 (83.8) |
| Overweight (25.0-29.9 kg/m^2^) | 1824 (4.0) | 2376(5.8) | 2973 (7.6) | 3176 (7.7) | 3827 (8.5) | 3210 (8.8) | 4036 (9.6) | 4366 (10.4) | 3661 (9.7) | 5395 (13.5) |
| Obesity (>30.0 kg/m^2^) | 470 (1.0) | 494 (1.2) | 623 (1.6) | 659 (1.6) | 758 (1.7) | 552 (1.5) | 778 (1.9) | 662 (1.6) | 541 (1.4) | 783 (2.0) |
| **Parental level of education, n (%)** |  |  |  |  |  |  |  |  |  |  |
| Compulsory school <9 years | 20 026 (44.1) | 17 083 (41.4) | 14 863 (38.0) | 14 579 (35.3) | 14 426 (31.9) | 10 761 (29.6) | 10 707 (25.5) | 9751 (23.3) | 7251 (19.1) | 6302 (15.8) |
| Secondary education | 18 756 (41.3) | 17 285 (41.8) | 16 845 (43.1) | 17 900 (43.4) | 20 141 (44.6) | 16 110 (44.4) | 19 218 (45.8) | 18 670 (44.6) | 17 198 (45.4) | 17 325 (43.5) |
| Post-secondary education <3 years | 2965 (6.5) | 2943 (7.1) | 3050 (7.8) | 3607 (8.7) | 4396 (9.7) | 3714 (10.2) | 4746 (11.3) | 5069 (12.1) | 5074 (13.4) | 5754 (14.4) |
| Post-secondary education >3 years | 3707 (8.2) | 4006 (9.7) | 4329 (11.1) | 5183 (12.6) | 6206 (13.7) | 5723 (15.8) | 7279 (17.4) | 8388 (20.0) | 8394 (22.1) | 10 468 (26.3) |
| **Parental highest income, n (%)** |  |  |  |  |  |  |  |  |  |  |
| Category 1 (low income) | 2525 (5.6) | 2152 (5.2) | 1834 (4.7) | 1837 (4.5) | 1830 (4.1) | 1429 (3.9) | 1497 (3.6) | 1289 (3.1) | 1047 (2.8) | 908 (2.3) |
| Category 2 | 5026 (11.1) | 4286 (10.4) | 3918 (10.0) | 3774 (9.1) | 4179 (9.3) | 3050 (8.4) | 3445 (8.2) | 3094 (7.4) | 2619 (6.9) | 2405 (6.0) |
| Category 3 | 11 782 (25.9) | 10 090 (24.4) | 9086 (23.3) | 9359 (22.7) | 10 032 (22.2) | 7564 (20.8) | 8613 (20.5) | 7890 (18.8) | 6899 (18.2) | 6672 (16.7) |
| Category 4 | 14 520 (31.9) | 12 762 (30.9) | 12 332 (31.6) | 12 914 (31.3) | 13 947 (30.9) | 11 247 (31.0) | 12 787 (30.5) | 12 586 (30.1) | 11 477 (30.3) | 11 658 (29.3) |
| Category 5 (high income) | 11 601 (25.5) | 12 027 (29.1) | 11 917 (30.5) | 13 385 (32.4) | 15 181 (33.6) | 13 018 (35.9) | 15 608 (37.2) | 17 019 (40.6) | 15 875 (41.9) | 18 206 (45.7) |
| IQR = interquartile range. SD = standard deviation. | | | | | | | | | | |

| **Supplemental table 2. Numbers censored due, death, emigration, and end of follow-up.** | | |
| --- | --- | --- |
|  | **Cohort analysis**   **(N=1 013 885)** | **Sibling analysis**   **(N=410 998)** |
| **Depression diagnosis** |  |  |
| Outcome | 47 283 (4.7) | 18 150 (4.4) |
| Death | 55 409 (5.5) | 21 227 (5.2) |
| Emigration | 65 043 (6.4) | 25 457 (6.2) |
| End of follow-up | 846 150 (83.5) | 345 364 (84.2) |
| **Antidepressive medications** |  |  |
| Outcome | 237 409 (23.4) | 93 150 (22.7) |
| Death | 43 380 (4.3) | 16 647 (4.1) |
| Emigration | 56 448 (5.6) | 22 237 (5.4) |
| End of follow-up | 676 648 (66.7) | 278 164 (67.8) |
| Number of events and numbers censored are shown as n (%). | | |

| **Supplemental table 3. Subtypes of depression diagnoses among individuals with a depression outcome during follow-up.** | | |
| --- | --- | --- |
| **Depression subtype, ICD-10 code** | **N cases in the full cohort** | **N cases in the sibling cohort** |
| Major depressive disorder, single episode, mild, F32.0 | 4801 | 1803 |
| Major depressive disorder, single episode, moderate, F32.1 | 11 570 | 4379 |
| Major depressive disorder, single episode, severe without psychotic features, F32.2 | 4697 | 1920 |
| Major depressive disorder, single episode, severe with psychotic features, F32.3 | 1271 | 531 |
| Major depressive disorder, single episode, in partial remission, F32.4 | 1 | 0 |
| Other depressive episodes, F32.8 | 615 | 253 |
| Major depressive disorder, single episode, unspecified, F32.9 | 24 444 | 9308 |

| **Supplemental table 4. Overlap between individuals with a depression diagnosis in the National Patient Register and individuals with a dispensation of antidepressants in the Prescribed Drug Register.** | | | |
| --- | --- | --- | --- |
|  | **No antidepressants** | **Antidepressants** | **Total** |
| **No depression diagnosis** | 772 214 | 194 388 | 966 602 |
| **Depression diagnosis** | 4262 | 43 021 | 47 283 |
| **Total** | 776 476 | 237 409 | 1 013 885 |
| Tetrachoric correlation: 0.78, *P*<0.00001 | | | |

| **Supplemental table 5. Unadjusted hazard ratios for depression diagnosis and dispensation of antidepressive medications by deciles of cardiorespiratory fitness in the full cohort.** | | | | | |
| --- | --- | --- | --- | --- | --- |
|  | **Depression diagnosis** | |  | **Dispensation of antidepressive medications** | |
| **Deciles  of fitness** | **Cases/N** | **HR (95% CI)** |  | **Cases/N** | **HR (95% CI)** |
| D1 | 6905/113 887 | Ref. |  | 32 125/113 887 | Ref. |
| D2 | 5392/100 857 | 0.87 (0.84, 0.90) |  | 26 175/100 857 | 0.90 (0.88, 0.91) |
| D3 | 5094/96 126 | 0.90 (0.87, 0.93) |  | 24 919/96 126 | 0.95 (0.94, 0.97) |
| D4 | 4985/100 065 | 0.87 (0.83, 0.90) |  | 24 668/100 065 | 0.93 (0.91, 0.95) |
| D5 | 5272/110 985 | 0.88 (0.85, 0.91) |  | 26 702/110 985 | 0.99 (0.97, 1.01) |
| D6 | 4019/88 231 | 0.85 (0.82, 0.89) |  | 20 319/88 231 | 0.96 (0.94, 0.98) |
| D7 | 4672/103 963 | 0.90 (0.87, 0.94) |  | 23 332/103 963 | 1.04 (1.02, 1.06) |
| D8 | 4164/102 896 | 0.81 (0.78, 0.85) |  | 21 379/102 896 | 0.96 (0.94, 0.98) |
| D9 | 3540/96 282 | 0.81 (0.78, 0.84) |  | 19 256/96 282 | 1.05 (1.03, 1.07) |
| D10 | 3240/100 593 | 0.72 (0.69, 0.76) |  | 18 534/100 593 | 0.99 (0.97, 1.01) |
| D = decile. CI = confidence interval. HR = hazard ratio. | | | | | |

| Supplemental table 6. Estimated preventable fraction of depression diagnosis and dispensation of antidepressive medications ****at 65 years of age associated with a moderate (shifting those below deciles 5 to decile 5), or an extreme hypothetical intervention (shifting everyone to decile 10), in cohort and sibling analysis.**** | | |
| --- | --- | --- |
|  | **Depression diagnosis** | |
|  | **Cohort analysis  (N=1 013 885)** | **Sibling analysis  (N=410 198)** |
| **Hypothetical intervention** | **Preventable fraction,  % (95% CI)** | **Preventable fraction,  % (95% CI)** |
| Moderate | 10.8 (8.6, 13.0) | 7.8 (2.8 to 12.7) |
| Extreme | 29.1 (26.7, 31.6) | 17.6 (11.0, 24.2) |
|  | **Dispensation of antidepressive medications** | |
|  | **Cohort analysis  (N=1 013 885)** | **Sibling analysis  (N=410 198)** |
| **Hypothetical intervention** | **Preventable fraction,  % (95% CI)** | **Preventable fraction,  % (95% CI)** |
| Moderate | 5.8 (5.0, 6.6) | 3.5 (1.7, 5.4) |
| Extreme | 17.8 (16.7, 18.6) | 10.4 (7.8, 13.0) |
| CI = confidence interval. The models were adjusted for age at conscription, year of conscription, body mass index, IQ, parental education, and parental income. | | |

| Supplemental table 7. ****Hazard ratios for depression diagnosis and dispensation of antidepressive medications by deciles of cardiorespiratory fitness in cohort and sibling analysis, with and without allowing for effect modification by overweight, and in strata of overweight status.**** | | | | | |
| --- | --- | --- | --- | --- | --- |
|  | **Assuming no effect modification by body mass index, as reported in the main article^a^** | |  | **Incorporation interaction terms between fitness and overweight^b^** | |
|  | **Cohort analysis  (N=1 013 885)** | **Sibling analysis  (N=410 198)** |  | **Cohort analysis  (N=1 013 885** | **Sibling analysis  (N=410 198)** |
| **Deciles of fitness** | **HR (95% CI)** | **HR (95% CI)** | **Deciles of fitness** | **HR (95% CI)** | **HR (95% CI)** |
| **Total population** | **Depression diagnosis** | | **Total population** | **Depression diagnosis** | |
| D1 | Ref. | Ref. | D1 | Ref. | Ref. |
| D2 | 0.91 (0.88, 0.95) | 0.89 (0.82, 0.97) | D2 | 0.91 (0.88, 0.94) | 0.89 (0.82, 0.97) |
| D3 | 0.89 (0.86, 0.93) | 0.87 (0.80, 0.94) | D3 | 0.89 (0.86, 0.92) | 0.86 (0.79, 0.94) |
| D4 | 0.84 (0.81, 0.88) | 0.89 (0.81, 0.97) | D4 | 0.85 (0.81, 0.88) | 0.89 (0.81, 0.97) |
| D5 | 0.79 (0.76, 0.82) | 0.82 (0.75, 0.90) | D5 | 0.77 (0.74, 0.80) | 0.81 (0.74, 0.89) |
| D6 | 0.76 (0.73, 0.79) | 0.79 (0.71, 0.86) | D6 | 0.76 (0.73, 0.79) | 0.78 (0.71, 0.86) |
| D7 | 0.74 (0.71, 0.77) | 0.81 (0.73, 0.89) | D7 | 0.73 (0.70, 0.76) | 0.80 (0.73, 0.89) |
| D8 | 0.68 (0.65, 0.71) | 0.71 (0.64, 0.78) | D8 | 0.66 (0.64, 0.69) | 0.69 (0.62, 0.76) |
| D9 | 0.61 (0.59, 0.64) | 0.70 (0.63, 0.78) | D9 | 0.60 (0.57, 0.63) | 0.68 (0.61, 0.76) |
| D10 | 0.54 (0.52, 0.57) | 0.67 (0.59, 0.75) | D10 | 0.53 (0.50, 0.55) | 0.64 (0.57, 0.72) |
|  |  |  | **Not overweight (<25.0 kg/m^2^)** |  |  |
|  |  |  | D1 | Ref. | Ref. |
|  |  |  | D2 | 0.91 (0.88, 0.94) | 0.89 (0.82, 0.97) |
|  |  |  | D3 | 0.89 (0.86, 0.92) | 0.86 (0.79, 0.94) |
|  |  |  | D4 | 0.84 (0.81, 0.88) | 0.89 (0.81, 0.97) |
|  |  |  | D5 | 0.77 (0.74, 0.80) | 0.81 (0.74, 0.89) |
|  |  |  | D6 | 0.76 (0.73, 0.79) | 0.78 (0.71, 0.86) |
|  |  |  | D7 | 0.73 (0.70, 0.76) | 0.80 (0.73, 0.89) |
|  |  |  | D8 | 0.66 (0.64, 0.69) | 0.69 (0.62, 0.76) |
|  |  |  | D9 | 0.60 (0.57, 0.63) | 0.68 (0.61, 0.76) |
|  |  |  | D10 | 0.53 (0.50, 0.55) | 0.64 (0.57, 0.72) |
|  |  |  | **Overweight >25.0 kg/m^2^)** |  |  |
|  |  |  | D1 | Ref. | Ref. |
|  |  |  | D2 | 0.95 (0.83, 1.08) | 0.84 (0.61, 1.15) |
|  |  |  | D3 | 0.92 (0.81, 1.05) | 0.87 (0.65, 1.17) |
|  |  |  | D4 | 0.83 (0.73, 0.94) | 0.82 (0.61, 1.11) |
|  |  |  | D5 | 0.91 (0.81, 1.03) | 0.82 (0.61, 1.09) |
|  |  |  | D6 | 0.80 (0.70, 0.91) | 0.76 (0.56, 1.02) |
|  |  |  | D7 | 0.80 (0.71, 0.90) | 0.74 (0.55, 0.99) |
|  |  |  | D8 | 0.76 (0.67, 0.86) | 0.78 (0.58, 1.04) |
|  |  |  | D9 | 0.71 (0.62, 0.81) | 0.75 (0.55, 1.03) |
|  |  |  | D10 | 0.63 (0.55, 0.71) | 0.68 (0.51, 0.92) |
| **Total population** | **Dispensation of antidepressive medications** | | **Total population** | **Dispensation of antidepressive medications** | |
| D1 | Ref. | Ref. | D1 | Ref. | Ref. |
| D2 | 0.92 (0.90, 0.93) | 0.94 (0.91, 0.98) | D2 | 0.93 (0.92, 0.94) | 0.95 (0.91, 0.98) |
| D3 | 0.92 (0.90, 0.93) | 0.93 (0.90, 0.97) | D3 | 0.93 (0.91, 0.94) | 0.94 (0.91, 0.97) |
| D4 | 0.87 (0.85, 0.88) | 0.91 (0.88, 0.95) | D4 | 0.88 (0.87, 0.90) | 0.92 (0.89, 0.96) |
| D5 | 0.84 (0.82, 0.85) | 0.89 (0.86, 0.93) | D5 | 0.85 (0.84, 0.86) | 0.90 (0.86, 0.93) |
| D6 | 0.80 (0.78, 0.81) | 0.85 (0.82, 0.89) | D6 | 0.82 (0.80, 0.83) | 0.86 (0.82, 0.89) |
| D7 | 0.77 (0.76, 0.79) | 0.85 (0.81, 0.88) | D7 | 0.79 (0.78, 0.80) | 0.86 (0.82, 0.89) |
| D8 | 0.72 (0.70, 0.73) | 0.81 (0.78, 0.85) | D8 | 0.74 (0.72, 0.75) | 0.82 (0.79, 0.86) |
| D9 | 0.69 (0.68, 0.70) | 0.79 (0.76, 0.83) | D9 | 0.71 (0.70, 0.72) | 0.80 (0.77, 0.84) |
| D10 | 0.63 (0.62, 0.65) | 0.76 (0.72, 0.80) | D10 | 0.66 (0.65, 0.67) | 0.77 (0.73, 0.80) |
|  |  |  | **Not overweight (<25.0 kg/m^2^)** |  |  |
|  |  |  | D1 | Ref. | Ref. |
|  |  |  | D2 | 0.93 (0.92, 0.94) | 0.95 (0.91, 0.98) |
|  |  |  | D3 | 0.93 (0.91, 0.94) | 0.94 (0.91, 0.97) |
|  |  |  | D4 | 0.88 (0.87, 0.90) | 0.92 (0.89, 0.96) |
|  |  |  | D5 | 0.85 (0.84, 0.86) | 0.90 (0.86, 0.93) |
|  |  |  | D6 | 0.82 (0.80, 0.83) | 0.86 (0.82, 0.89) |
|  |  |  | D7 | 0.79 (0.78, 0.80) | 0.86 (0.82, 0.89) |
|  |  |  | D8 | 0.74 (0.72, 0.75) | 0.82 (0.79, 0.86) |
|  |  |  | D9 | 0.71 (0.70, 0.72) | 0.80 (0.77, 0.84) |
|  |  |  | D10 | 0.66 (0.64, 0.67) | 0.77 (0.73, 0.80) |
|  |  |  | **Overweight >25.0 kg/m^2^)** |  |  |
|  |  |  | D1 | Ref. | Ref. |
|  |  |  | D2 | 0.91 (0.86, 0.96) | 0.98 (0.86, 1.12) |
|  |  |  | D3 | 0.92 (0.87, 0.97) | 0.92 (0.81, 1.05) |
|  |  |  | D4 | 0.87 (0.82, 0.92) | 0.88 (0.78, 1.00) |
|  |  |  | D5 | 0.89 (0.84, 0.93) | 0.93 (0.83, 1.06) |
|  |  |  | D6 | 0.85 (0.81, 0.90) | 0.90 (0.79, 1.02) |
|  |  |  | D7 | 0.83 (0.79, 0.87) | 0.85 (0.75, 0.97) |
|  |  |  | D8 | 0.78 (0.74, 0.82) | 0.81 (0.71, 0.92) |
|  |  |  | D9 | 0.76 (0.72, 0.80) | 0.83 (0.72, 0.94) |
|  |  |  | D10 | 0.69 (0.65, 0.72) | 0.82 (0.72, 0.92) |
| CI = confidence interval. D = decile. HR = hazard ratio.  ^a^Adjusted for age at conscription, year of conscription, IQ, body mass index, parental education, and parental income. ^b^Adjusted for age at conscription, year of conscription, IQ, body mass indexz, parental education, parental income (and additionally for interaction terms between fitness and overweight status in the total population). | | | | | |

| **Supplemental table 8. Hazard ratios for depression diagnosis and dispensation of antidepressive medications by deciles of cardiorespiratory fitness in cohort analysis (as reported in the main article), in the sibling cohort using standard analysis, and using sibling analysis (as reported in the main article)** | | | |
| --- | --- | --- | --- |
|  | **Depression diagnosis** | | |
|  | **Cohort analysis as reported in the main article (N=1 013 885)** | **Standard analysis replicated in the sibling cohort (N=410 198)** | **Sibling analysis as reported in the main article (N=410 198)** |
| **Deciles of fitness** | **HR (95% CI)** | **HR (95% CI)** | **HR (95% CI)** |
| D1 | Ref. | Ref. | Ref. |
| D2 | 0.91 (0.88, 0.95) | 0.90 (0.85, 0.95) | 0.89 (0.82, 0.97) |
| D3 | 0.89 (0.86, 0.93) | 0.88 (0.83, 0.94) | 0.87 (0.80, 0.94) |
| D4 | 0.84 (0.81, 0.88) | 0.84 (0.79, 0.90) | 0.89 (0.81, 0.97) |
| D5 | 0.79 (0.76, 0.82) | 0.78 (0.73, 0.83) | 0.82 (0.75, 0.90) |
| D6 | 0.76 (0.73, 0.79) | 0.76 (0.71, 0.81) | 0.79 (0.71, 0.86) |
| D7 | 0.74 (0.71, 0.77) | 0.72 (0.68, 0.77) | 0.81 (0.73, 0.89) |
| D8 | 0.68 (0.65, 0.71) | 0.66 (0.62, 0.71) | 0.71 (0.64, 0.78) |
| D9 | 0.61 (0.59, 0.64) | 0.61 (0.57, 0.66) | 0.70 (0.63, 0.78) |
| D10 | 0.54 (0.52, 0.57) | 0.57 (0.53, 0.62) | 0.67 (0.59, 0.75) |
|  | **Dispensation of antidepressive medications** | | |
|  | **Cohort analysis as reported in the main article (N=1 013 885)** | **Standard analysis replicated in the sibling cohort (N=410 198)** | **Sibling analysis as reported in the main article (N=410 198)** |
| **Deciles of fitness** | **HR (95% CI)** | **HR (95% CI)** | **HR (95% CI)** |
| D1 | Ref. | Ref. | Ref. |
| D2 | 0.92 (0.90, 0.93) | 0.93 (0.90, 0.95) | 0.94 (0.91, 0.98) |
| D3 | 0.92 (0.90, 0.93) | 0.92 (0.90, 0.95) | 0.93 (0.90, 0.97) |
| D4 | 0.87 (0.85, 0.88) | 0.86 (0.85, 0.88) | 0.91 (0.88, 0.95) |
| D5 | 0.84 (0.82, 0.85) | 0.84 (0.84, 0.87) | 0.89 (0.86, 0.93) |
| D6 | 0.80 (0.78, 0.81) | 0.81 (0.78, 0.83) | 0.85 (0.82, 0.89) |
| D7 | 0.77 (0.76, 0.79) | 0.77 (0.75, 0.79) | 0.85 (0.81, 0.88) |
| D8 | 0.72 (0.70, 0.73) | 0.72 (0.70, 0.75) | 0.81 (0.78, 0.85) |
| D9 | 0.69 (0.68, 0.70) | 0.69 (0.67, 0.71) | 0.79 (0.76, 0.83) |
| D10 | 0.63 (0.62, 0.65) | 0.65 (0.63, 0.68) | 0.76 (0.72, 0.80) |
| CI = confidence interval. D = decile. HR = hazard ratio. The models were adjusted for age at conscription, year of conscription, body mass index, IQ, parental education, and parental income. | | | |

| **Supplemental table 9. Hazard ratios for depression diagnosis and dispensation of antidepressive medications by deciles of cardiorespiratory fitness in cohort and sibling analysis, restricted to those who conscribed year 1985 or later.** | | |
| --- | --- | --- |
|  | **Depression diagnosis** | |
|  | **Cohort analysis  (N=439 888)** | **Sibling analysis  (N=119 322)** |
| **Deciles of fitness** | **HR (95% CI)** | **HR (95% CI)** |
| D1 | Ref. | Ref. |
| D2 | 0.87 (0.83, 0.91) | 1.01 (0.78, 1.31) |
| D3 | 0.77 (0.74, 0.81) | 0.78 (0.62, 0.99) |
| D4 | 0.70 (0.67, 0.73) | 0.81 (0.64, 1.03) |
| D5 | 0.64 (0.61, 0.68) | 0.90 (0.73, 1.11) |
| D6 | 0.58 (0.55, 0.61) | 0.79 (0.64, 0.99) |
| D7 | 0.55 (0.52, 0.58) | 0.87 (0.70, 1.08) |
| D8 | 0.51 (0.48, 0.53) | 0.70 (0.56, 0.87) |
| D9 | 0.46 (0.44, 0.49) | 0.67 (0.54, 0.84) |
| D10 | 0.39 (0.37, 0.41) | 0.65 (0.52, 0.81) |
|  | **Dispensation of antidepressive medications** | |
|  | **Cohort analysis  (N=439 888)** | **Sibling analysis  (N=119 322)** |
| **Deciles of fitness** | **HR (95% CI)** | **HR (95% CI)** |
| D1 | Ref. | Ref. |
| D2 | 0.91 (0.88, 0.95) | 0.93 (0.83, 1.06) |
| D3 | 0.89 (0.85, 0.92) | 0.95 (0.85, 1.06) |
| D4 | 0.86 (0.83, 0.89) | 0.94 (0.84, 1.05) |
| D5 | 0.81 (0.78, 0.84) | 0.89 (0.81, 0.99) |
| D6 | 0.77 (0.74, 0.79) | 0.87 (0.78, 0.96) |
| D7 | 0.73 (0.71, 0.76) | 0.85 (0.77, 0.93) |
| D8 | 0.68 (0.65, 0.70) | 0.81 (0.73, 0.89) |
| D9 | 0.65 (0.63, 0.67) | 0.75 (0.68, 0.84) |
| D10 | 0.59 (0.57, 0.61) | 0.73 (0.66, 0.81) |
| CI = confidence interval. D = decile. HR = hazard ratio.  The models were adjusted for age at conscription, year of conscription, body mass index, IQ, parental education, and parental income. | | |

| **Supplemental table 10. Hazard ratios for depression diagnosis and dispensation of antidepressive medications by deciles of cardiorespiratory fitness in cohort and sibling analysis, modelling the covariate year of conscription using restricted cubic splines versus as a categorical variable as in the main analysis.** | | | | | |
| --- | --- | --- | --- | --- | --- |
|  | | **Depression diagnosis** | | | |
|  | **Cohort analysis (N=1 013 885)** | | | **Sibling analysis (N=410 198)** | |
|  | **Modelling year of conscription as a categorial variable** | | **Modelling year of conscription using restricted cubic splines^a^** | **Modelling year of conscription as a categorial variable** | **Modelling year of conscription using restricted cubic splines^a^** |
| **Deciles of fitness** | **HR (95% CI)** | | **HR (95% CI)** | **HR (95% CI)** | **HR (95% CI)** |
| D1 | Ref. | | Ref. | Ref. | Ref. |
| D2 | 0.91 (0.88, 0.95) | | 0.91 (0.88, 0.95) | 0.89 (0.82, 0.97) | 0.89 (0.82, 0.97) |
| D3 | 0.89 (0.86, 0.93) | | 0.89 (0.86, 0.92) | 0.87 (0.80, 0.94) | 0.86 (0.79, 0.94) |
| D4 | 0.84 (0.81, 0.88) | | 0.84 (0.81, 0.87) | 0.89 (0.81, 0.97) | 0.88 (0.81, 0.96) |
| D5 | 0.79 (0.76, 0.82) | | 0.78 (0.75, 0.81) | 0.82 (0.75, 0.90) | 0.81 (0.74, 0.88) |
| D6 | 0.76 (0.73, 0.79) | | 0.76 (0.73, 0.79) | 0.79 (0.71, 0.86) | 0.77 (0.70, 0.85) |
| D7 | 0.74 (0.71, 0.77) | | 0.73 (0.70, 0.76) | 0.81 (0.73, 0.89) | 0.79 (0.72, 0.87) |
| D8 | 0.68 (0.65, 0.71) | | 0.67 (0.64, 0.70) | 0.71 (0.64, 0.78) | 0.69 (0.63, 0.76) |
| D9 | 0.61 (0.59, 0.64) | | 0.60 (0.58, 0.63) | 0.70 (0.63, 0.78) | 0.68 (0.61, 0.75) |
| D10 | 0.54 (0.52, 0.57) | | 0.53 (0.51, 0.56) | 0.67 (0.59, 0.75) | 0.64 (0.57, 0.72) |
|  | **Dispensation of antidepressive medications** | | | | |
|  | **Cohort analysis (N=1 013 885)** | | | **Sibling analysis (N=410 198)** | |
|  | **Modelling year of conscription as a categorial variable** | | **Modelling year of conscription using restricted cubic splines^a^** | **Modelling year of conscription as a categorial variable** | **Modelling year of conscription using restricted cubic splines^a^** |
| **Deciles of fitness** | **HR (95% CI)** | | **HR (95% CI)** | **HR (95% CI)** | **HR (95% CI)** |
| D1 | Ref. | | Ref. | Ref. | Ref. |
| D2 | 0.92 (0.90, 0.93) | | 0.92 (0.91, 0.94) | 0.94 (0.91, 0.98) | 0.95 (0.91, 0.98) |
| D3 | 0.92 (0.90, 0.93) | | 0.91 (0.90, 0.93) | 0.93 (0.90, 0.97) | 0.93 (0.89, 0.96) |
| D4 | 0.87 (0.85, 0.88) | | 0.86 (0.85, 0.88) | 0.91 (0.88, 0.95) | 0.91 (0.87, 0.94) |
| D5 | 0.84 (0.82, 0.85) | | 0.83 (0.81, 0.84) | 0.89 (0.86, 0.93) | 0.88 (0.85, 0.91) |
| D6 | 0.80 (0.78, 0.81) | | 0.79 (0.78, 0.81) | 0.85 (0.82, 0.89) | 0.84 (0.80, 0.87) |
| D7 | 0.77 (0.76, 0.79) | | 0.76 (0.75, 0.77) | 0.85 (0.81, 0.88) | 0.82 (0.79, 0.86) |
| D8 | 0.72 (0.70, 0.73) | | 0.70 (0.69, 0.72) | 0.81 (0.78, 0.85) | 0.79 (0.75, 0.82) |
| D9 | 0.69 (0.68, 0.70) | | 0.67 (0.66, 0.69) | 0.79 (0.76, 0.83) | 0.76 (0.73, 0.80) |
| D10 | 0.63 (0.62, 0.65) | | 0.60 (0.60, 0.63) | 0.76 (0.72, 0.80) | 0.72 (0.69, 0.76) |
| ^a^Knots placed at the 5^th^, 35^th^, 65^th^, and 95^th^ percentile.  CI = confidence interval. D = decile. HR = hazard ratio.  The models were adjusted for age at conscription, year of conscription (as indicated above), body mass index, IQ, parental education, and parental income. | | | | | |

| **Supplemental table 11. Hazard ratios for depression diagnosis and dispensation of antidepressive medications by deciles of cardiorespiratory fitness in cohort analysis, including regression adjustment for year of conscription as in the main analysis versus using stratified Cox regression conditioned on year of conscription.** | | | | | |
| --- | --- | --- | --- | --- | --- |
|  | | **Depression diagnosis** | | | |
|  | **Depression diagnosis** | | | **Dispensation of antidepressive medications** | |
|  | **Adjusting for year of conscription as per main analysis** | | **Stratified Cox regression conditioned on year of conscription** | **Adjusting for year of conscription as per main analysis** | **Stratified Cox regression conditioned on year of conscription** |
| **Deciles of fitness** | **HR (95% CI)** | | **HR (95% CI)** | **HR (95% CI)** | **HR (95% CI)** |
| D1 | Ref. | | Ref. | Ref. | Ref. |
| D2 | 0.91 (0.88, 0.95) | | 0.91 (0.88, 0.95) | 0.92 (0.90, 0.93) | 0.92 (0.90, 0.93) |
| D3 | 0.89 (0.86, 0.93) | | 0.89 (0.86, 0.92) | 0.92 (0.90, 0.93) | 0.91 (0.90, 0.93) |
| D4 | 0.84 (0.81, 0.88) | | 0.84 (0.81, 0.87) | 0.87 (0.85, 0.88) | 0.86 (0.85, 0.87) |
| D5 | 0.79 (0.76, 0.82) | | 0.78 (0.75, 0.81) | 0.84 (0.82, 0.85) | 0.82 (0.81, 0.84) |
| D6 | 0.76 (0.73, 0.79) | | 0.75 (0.72, 0.79) | 0.80 (0.78, 0.81) | 0.79 (0.77, 0.80) |
| D7 | 0.74 (0.71, 0.77) | | 0.73 (0.70, 0.76) | 0.77 (0.76, 0.79) | 0.76 (0.74, 0.77) |
| D8 | 0.68 (0.65, 0.71) | | 0.67 (0.64, 0.70) | 0.72 (0.70, 0.73) | 0.70 (0.69, 0.72) |
| D9 | 0.61 (0.59, 0.64) | | 0.60 (0.57, 0.63) | 0.69 (0.68, 0.70) | 0.67 (0.66, 0.68) |
| D10 | 0.54 (0.52, 0.57) | | 0.53 (0.51, 0.56) | 0.63 (0.62, 0.65) | 0.62 (0.60, 0.63) |
| CI = confidence interval. D = decile. HR = hazard ratio.  The models were adjusted for age at conscription, year of conscription (as indicated above), body mass index, IQ, parental education, and parental income. | | | | | |

| **Supplemental table 12. Standardised cumulative incidences of depression diagnosis and dispensation of antidepressive medications at 65 years of age by deciles of cardiorespiratory fitness in cohort and sibling analysis, allowing the effect of fitness to vary across follow-up time^a^.** | | | | | | | |  |
| --- | --- | --- | --- | --- | --- | --- | --- | --- |
|  | **Depression diagnosis** | | | | | | |  |
|  | **Cohort analysis  (N=1 013 885)** | |  | | **Sibling analysis  (N=410 198)** | | |  |
| **Deciles of fitness** | **Incidence at age 65, % (95% CI)** | **Incidence  difference, % (95% CI)** | |  | | **Incidence at age 65, % (95% CI)** | **Incidence  difference, % (95% CI)** | |
| D1 | 8.7 (8.5, 9.0) | Ref. | |  | | 7.5 (7.0, 7.9) | Ref. | |
| D2 | 8.0 (7.8, 8.3) | -0.7 (-1.0, -0.4) | |  | | 6.6 (6.2, 7.1) | -0.8 (-1.4, -0.3) | |
| D3 | 7.9 (7.6, 8.1) | -0.9 (-1.2, -0.6) | |  | | 6.6 (6.2, 7.0) | -0.9 (-1.5, -0.3) | |
| D4 | 7.4 (7.2, 7.6) | -1.4 (-1.7, -1.1) | |  | | 6.6 (6.2, 7.0) | -0.8 (-1.5, -0.2) | |
| D5 | 6.8 (6.6, 7.2) | -1.9 (-2.2, -1.6) | |  | | 6.2 (5.8, 6.6) | -1.2 (-1.8, -0.6) | |
| D6 | 6.6 (6.4, 6.9) | -2.1 (-2.4, 1.8) | |  | | 5.7 (5.3, 6.2) | -1.7 (-2.4, -1.1) | |
| D7 | 6.2 (6.0, 6.4) | -2.5 (-2.9, -2.2) | |  | | 5.9 (5.4, 6.3) | -1.6 (-2.3, -0.9) | |
| D8 | 5.8 (5.6, 6.0) | -3.0 (-3.3, -2.7) | |  | | 5.4 (5.0, 5.8) | -2.1 (-2.7, -1.4) | |
| D9 | 5.1 (4.8, 5.3) | -3.7 (-4.0, -3.3) | |  | | 5.1 (4.6, 5.6) | -2.4 (-3.1, -1.6) | |
| D10 | 4.5 (4.2, 4.7) | -4.3 (-4.6, -3.9) | |  | | 4.6 (4.1, 5.1) | -2.9 (-3.7, -2.1) | |
|  | **Dispensation of antidepressive medications** | | | | | | |  |
|  | **Cohort analysis  (N=1 013 885)** | |  | | **Sibling analysis  (N=410 198)** | | |  |
| **Deciles of fitness** | **Incidence at age 65, % (95% CI)** | **Incidence  difference, % (95% CI)** | |  | | **Incidence at age 65, % (95% CI)** | **Incidence  difference, % (95% CI)** | |
| D1 | 42.5 (42.1, 42.9) | Ref. | |  | | 37.2 (36.3, 38.1) | Ref. | |
| D2 | 40.1 (39.7, 40.5) | -2.4 (-2.9, -1.9) | |  | | 35.7 (34.8, 36.5) | -1.5 (-2.6, -0.5) | |
| D3 | 39.7 (39.3, 40.1) | -2.9 (-3.4, -2.3) | |  | | 35.2 (34.4, 36.1) | -2.0 (-3.1, -0.9) | |
| D4 | 38.2 (37.8, 38.6) | -4.4 (4.9, -3.8) | |  | | 35.0 (34.2, 35.9) | -2.2 (-3.4, -1.0) | |
| D5 | 36.8 (36.4, 37.2) | -5.7 (-6.3, -5.2) | |  | | 34.1 (33.3, 35.0) | -3.1 (-4.3, -1.9) | |
| D6 | 35.6 (35.1, 36.0) | -7.0 (-7.6, -6.4) | |  | | 32.7 (31.8, 33.7) | -4.5 (-5.8, -3.2) | |
| D7 | 34.1 (33.6, 34.6) | -8.5 (-9.1, -7.8) | |  | | 32.6 (31.7, 33.6) | -4.6 (-5.9, -3.2) | |
| D8 | 32.3 (31.8, 32.8) | -10.3 (-10.9, -9.6) | |  | | 31.6 (30.6, 32.6) | -5.6 (-7.0, -4.2) | |
| D9 | 30.6 (30.0, 31.3) | -11.9 (-12.7, -11.2) | |  | | 31.1 (29.9, 32.4) | -6.1 (-7.7, -4.4) | |
| D10 | 28.9 (28.2, 29.6) | -13.6 (-14.5, -12.8) | |  | | 30.3 (28.9, 31.7) | -6.9 (-8.8, -5.1) | |
| ^a^The incidences were allowed to vary across time using an interaction between a restricted cubic spline with three degrees of freedom of the follow-up time (centile 33 and 67 of the distribution of the uncensored log survival times) and the fitness deciles.  CI = confidence interval. D = decile. HR = hazard ratio. The models were adjusted for age at conscription, year of conscription, body mass index, IQ, parental education, and parental income, and cardiorespiratory fitness was modelled as a time-dependent exposure. | | | | | | | |  |

| **Supplemental table 13. Hazard ratios for depression diagnosis and dispensation of antidepressive medications by deciles of cardiorespiratory fitness expressed as Wmax (as reported in the main article), Wmax/kg, or estimated VO_2_max in cohort and sibling analysis.** | | | | | | |
| --- | --- | --- | --- | --- | --- | --- |
|  | **Depression diagnosis** | | | | | |
|  | **Wmax (as reported in main article)** | | **Wmax/kg** | | **Estimated VO_2_max** | |
|  | **Cohort analysis (N=1 013 885)** | **Sibling analysis (N=410 198)** | **Cohort analysis (N=1 013 885)** | **Sibling analysis (N=410 198)** | **Cohort analysis (N=1 013 885)** | **Sibling analysis (N=410 198)** |
| **Deciles of fitness** | **HR (95% CI)** | **HR (95% CI)** | **HR (95% CI)** | **HR (95% CI)** | **HR (95% CI)** | **HR (95% CI)** |
| D1 | Ref. | Ref. | Ref. | Ref. | Ref. | Ref. |
| D2 | 0.91 (0.88, 0.95) | 0.89 (0.82, 0.97) | 0.90 (0.86, 0.93) | 0.87 (0.80, 0.95) | 0.90 (0.86, 0.93) | 0.87 (0.80, 0.95) |
| D3 | 0.89 (0.86, 0.93) | 0.87 (0.80, 0.94) | 0.85 (0.82, 0.89) | 0.89 (0.81, 0.97) | 0.85 (0.82, 0.89) | 0.89 (0.81, 0.97) |
| D4 | 0.84 (0.81, 0.88) | 0.89 (0.81, 0.97) | 0.83 (0.79, 0.86) | 0.83 (0.75, 0.91) | 0.83 (0.79, 0.86) | 0.83 (0.75, 0.91) |
| D5 | 0.79 (0.76, 0.82) | 0.82 (0.75, 0.90) | 0.80 (0.77, 0.83) | 0.82 (0.75, 0.90) | 0.80 (0.77, 0.83) | 0.82 (0.75, 0.90) |
| D6 | 0.76 (0.73, 0.79) | 0.79 (0.71, 0.86) | 0.76 (0.73, 0.79) | 0.79 (0.72, 0.87) | 0.76 (0.73, 0.79) | 0.79 (0.72, 0.87) |
| D7 | 0.74 (0.71, 0.77) | 0.81 (0.73, 0.89) | 0.73 (0.70, 0.76) | 0.73 (0.66, 0.81) | 0.73 (0.70, 0.76) | 0.73 (0.66, 0.81) |
| D8 | 0.68 (0.65, 0.71) | 0.71 (0.64, 0.78) | 0.69 (0.66, 0.72) | 0.76 (0.69, 0.85) | 0.69 (0.66, 0.72) | 0.76 (0.69, 0.85) |
| D9 | 0.61 (0.59, 0.64) | 0.70 (0.63, 0.78) | 0.65 (0.62, 0.68) | 0.70 (0.63, 0.78) | 0.65 (0.62, 0.68) | 0.70 (0.63, 0.78) |
| D10 | 0.54 (0.52, 0.57) | 0.67 (0.59, 0.75) | 0.57 (0.54, 0.60) | 0.69 (0.61, 0.78) | 0.57 (0.54, 0.60) | 0.69 (0.61, 0.78) |
| **Cardiorespiratory fitness, per 1 MET** |  |  |  |  | 0.93 (0.93, 0.94) | 0.95 (0.94, 0.96) |
|  | **Dispensation of antidepressive medications** | | | | | |
|  | **Wmax (as reported in main article)** | | **Wmax/kg** | | **Estimated VO_2_max** | |
|  | **Cohort analysis (N=1 013 885)** | **Sibling analysis (N=410 198)** | **Cohort analysis (N=1 013 885)** | **Sibling analysis (N=410 198)** | **Cohort analysis (N=1 013 885)** | **Sibling analysis (N=410 198)** |
| **Deciles of fitness** | **HR (95% CI)** | **HR (95% CI)** | **HR (95% CI)** | **HR (95% CI)** | **HR (95% CI)** | **HR (95% CI)** |
| D1 | Ref. | Ref. | Ref. | Ref. | Ref. | Ref. |
| D2 | 0.92 (0.90, 0.93) | 0.94 (0.91, 0.98) | 0.91 (0.90, 0.93) | 0.95 (0.92, 0.99) | 0.91 (0.90, 0.93) | 0.95(0.92, 0.99) |
| D3 | 0.92 (0.90, 0.93) | 0.93 (0.90, 0.97) | 0.88 (0.87, 0.90) | 0.92 (0.89, 0.96) | 0.88 (0.87, 0.90) | 0.92 (0.89, 0.96) |
| D4 | 0.87 (0.85, 0.88) | 0.91 (0.88, 0.95) | 0.84 (0.83, 0.86) | 0.89 (0.86, 0.93) | 0.84 (0.83, 0.86) | 0.89 (0.86, 0.93) |
| D5 | 0.84 (0.82, 0.85) | 0.89 (0.86, 0.93) | 0.81 (0.79, 0.82) | 0.86 (0.82, 0.90) | 0.81 (0.79, 0.82) | 0.86 (0.82, 0.90) |
| D6 | 0.80 (0.78, 0.81) | 0.85 (0.82, 0.89) | 0.80 (0.79, 0.82) | 0.86 (0.82, 0.90) | 0.80 (0.79, 0.82) | 0.86 (0.82, 0.90) |
| D7 | 0.77 (0.76, 0.79) | 0.85 (0.81, 0.88) | 0.76 (0.74, 0.77) | 0.85 (0.81, 0.89) | 0.76 (0.74, 0.77) | 0.85 (0.81, 0.89) |
| D8 | 0.72 (0.70, 0.73) | 0.81 (0.78, 0.85) | 0.73 (0.71, 0.74) | 0.82 (0.79, 0.86) | 0.73 (0.71, 0.74) | 0.82 (0.79, 0.86) |
| D9 | 0.69 (0.68, 0.70) | 0.79 (0.76, 0.83) | 0.69 (0.68, 0.70) | 0.79 (0.75, 0.83) | 0.69 (0.68, 0.70) | 0.79 (0.75, 0.83) |
| D10 | 0.63 (0.62, 0.65) | 0.76 (0.72, 0.80) | 0.64 (0.63, 0.65) | 0.78 (0.74, 0.82) | 0.64 (0.63, 0.65) | 0.78 (0.74, 0.82) |
| **Cardiorespiratory fitness, per 1 MET** |  |  |  |  | 0.94 (0.94, 0.94) | 0.96 (0.96, 0.97) |
| CI = confidence interval. D = decile. HR = hazard ratio. MET = metabolic equivalent of task, computed from dividing estimated VO_2_max/ 3.5.  The models were adjusted for age at conscription, year of conscription, body mass index, IQ, parental education, and parental income. | | | | | | |

| **Supplemental table 14. Hazard ratios for depression diagnosis (F32 and F33) by deciles of cardiorespiratory fitness in cohort and sibling analysis.** | | | | |
| --- | --- | --- | --- | --- |
|  | **Depression diagnosis** | | | |
|  | **Cohort analysis  (N=1 013 885)** | | **Sibling analysis  (N=410 198)** | |
| **Deciles of fitness** | **Cases/N** | **HR (95% CI)** | **Cases/N** | **HR (95% CI)** |
| D1 | 8112/113 887 | Ref. | 3098/45 454 | Ref. |
| D2 | 6282/100 857 | 0.90 (0.87, 0.93) | 2447/41 317 | 0.90 (0.84, 0.97) |
| D3 | 5970/96 126 | 0.89 (0.86, 0.92) | 2301/39 087 | 0.89 (0.82, 0.96) |
| D4 | 5864/100 065 | 0.84 (0.81, 0.87) | 2292/41 269 | 0.90 (0.83, 0.98) |
| D5 | 6162/110 985 | 0.77 (0.75, 0.80) | 2357/45 169 | 0.84 (0.78, 0.91) |
| D6 | 4767/88 231 | 0.76 (0.73, 0.79) | 1835/36 308 | 0.80 (0.74, 0.88) |
| D7 | 5566/103 963 | 0.73 (0.71, 0.76) | 2047/41 950 | 0.83 (0.76, 0.91) |
| D8 | 4943/102 896 | 0.67 (0.64, 0.70) | 1838/41 878 | 0.73 (0.66, 0.80) |
| D9 | 4200/96 282 | 0.60 (0.58, 0.63) | 1547/37 917 | 0.72 (0.65, 0.80) |
| D10 | 3875/100 593 | 0.53 (0.51, 0.56) | 1510/39 849 | 0.69 (0.62, 0.77) |
| CI = confidence interval. D = decile. HR = hazard ratio. The models were adjusted for age at conscription, year of conscription, body mass index, IQ, parental education, and parental income. | | | | |

| **Supplemental table 15. Hazard ratios for a first versus second dispensation of antidepressive medications by deciles of cardiorespiratory fitness in cohort and sibling analysis.** | | | | |
| --- | --- | --- | --- | --- |
|  | **First dispensation of antidepressive medications** | | | |
|  | **Cohort analysis  (N=1 013 885)** | | **Sibling analysis  (N=410 198)** | |
| **Deciles of fitness** | **Cases/N** | **HR (95% CI)** | **Cases/N** | **HR (95% CI)** |
| D1 | 32 125/113 887 | Ref. | 12 512/45 454 | Ref. |
| D2 | 26 175/100 857 | 0.92 (0.90, 0.93) | 10 516/41 317 | 0.94 (0.91, 0.98) |
| D3 | 24 919/96 126 | 0.92 (0.90, 0.93) | 9936/39 087 | 0.93 (0.90, 0.97) |
| D4 | 24 668/100 065 | 0.87 (0.85, 0.88) | 9786/41 269 | 0.91 (0.88, 0.95) |
| D5 | 26 702/110 985 | 0.84 (0.82, 0.85) | 10 556/45 169 | 0.89 (0.86, 0.93) |
| D6 | 20 319/88 231 | 0.80 (0.78, 0.81) | 8081/36 308 | 0.85 (0.82, 0.89) |
| D7 | 23 332/103 963 | 0.77 (0.76, 0.79) | 8969/41 950 | 0.85 (0.81, 0.88) |
| D8 | 21 379/102 896 | 0.72 (0.70, 0.73) | 8409/41 878 | 0.81 (0.78, 0.85) |
| D9 | 19 256/96 282 | 0.69 (0.68, 0.70) | 7231/27 243 | 0.79 (0.76, 0.83) |
| D10 | 18 534/100 593 | 0.63 (0.62, 0.65) | 7154/39 849 | 0.76 (0.72, 0.80) |
|  | **Second dispensation of antidepressive medications** | | | |
|  | **Cohort analysis  (N=1 013 885)** | | **Sibling analysis  (N=410 198)** | |
| **Deciles of fitness** | **Cases/N** | **HR (95% CI)** | **Cases/N** | **HR (95% CI)** |
| D1 | 25 402/113 887 | Ref. | 9903/45 454 | Ref. |
| D2 | 20 585/100 857 | 0.92 (0.90, 0.93) | 8201/41 317 | 0.93 (0.89, 0.97) |
| D3 | 19 744/96 126 | 0.91 (0.90, 0.93) | 7845/39 087 | 0.93 (0.89, 0.97) |
| D4 | 19 465/100 065 | 0.86 (0.84, 0.88) | 7722/41 269 | 0.90 (0.87, 0.94) |
| D5 | 21 238/110 985 | 0.83 (0.82, 0.85) | 8359/45 169 | 0.88 (0.84, 0.92) |
| D6 | 16 228/88 231 | 0.80 (0.78, 0.81) | 6459/36 308 | 0.84 (0.80, 0.88) |
| D7 | 18 686/103 963 | 0.77 (0.75, 0.78) | 7066/41 950 | 0.83 (0.79, 0.87) |
| D8 | 17 023/102 896 | 0.71 (0.69, 0.72) | 6605/41 878 | 0.79 (0.75, 0.83) |
| D9 | 15 429/96 282 | 0.68 (0.67, 0.70) | 5741/27 243 | 0.77 (0.73, 0.81) |
| D10 | 14 901/100 593 | 0.63 (0.61, 0.64) | 5731/39 849 | 0.75 (0.71, 0.79) |
| CI = confidence interval. D = decile. HR = hazard ratio. The models were adjusted for age at conscription, year of conscription, body mass index, IQ, parental education, and parental income. | | | | |

| **Supplemental table 16. Hazard ratios for depression diagnosis and dispensation of antidepressive medications by deciles of cardiorespiratory fitness in cohort and sibling analysis, excluding everyone with pre-existing psychiatric disorders or symptoms at baseline.** | | | | |
| --- | --- | --- | --- | --- |
|  | **Depression diagnosis** | | | |
|  | **Cohort analysis  (N=960 374)** | | **Sibling analysis  (N=375 222)** | |
| **Deciles of fitness** | **Cases/N** | **HR (95% CI)** | **Cases/N** | **HR (95% CI)** |
| D1 | 5585/99 031 | Ref. | 2016/37 503 | Ref. |
| D2 | 4645/92 299 | 0.92 (0.88, 0.96) | 1698/35 954 | 0.90 (0.82, 0.99) |
| D3 | 4414/89 100 | 0.90 (0.86, 0.93) | 1609/34 670 | 0.87 (0.79, 0.95) |
| D4 | 4498/94 488 | 0.88 (0.83, 0.90) | 1690/37 448 | 0.90 (0.82, 0.99) |
| D5 | 4863/105 987 | 0.81 (0.78, 0.85) | 1775/41 624 | 0.82 (0.74, 0.90) |
| D6 | 3744/85 001 | 0.79 (0.75, 0.82) | 1389/33 916 | 0.80 (0.72, 0.89) |
| D7 | 4393/100 627 | 0.77 (0.73, 0.80) | 1554/39 490 | 0.81 (0.73, 0.90) |
| D8 | 3991/100 446 | 0.71 (0.68, 0.74) | 1448/39 850 | 0.73 (0.66, 0.81) |
| D9 | 3390/94 375 | 0.64 (0.61, 0.68) | 1214/36 309 | 0.72 (0.64, 0.81) |
| D10 | 3155/99 020 | 0.57 (0.54, 0.60) | 1194/38 458 | 0.69 (0.61, 0.78) |
|  | **Dispensation of antidepressive medications** | | | |
|  | **Cohort analysis  (N=960 374)** | | **Sibling analysis  (N=375 222)** | |
| **Deciles of fitness** | **Cases/N** | **HR (95% CI)** | **Cases/N** | **HR (95% CI)** |
| D1 | 26 587/99 031 | Ref. | 9729/37 503 | Ref. |
| D2 | 23 145/92 299 | 0.94 (0.92, 0.95) | 8734/35 954 | 0.95 (0.92, 0.99) |
| D3 | 22 329/89 100 | 0.93 (0.91, 0.95) | 8449/34 670 | 0.94 (0.90, 0.98) |
| D4 | 22 647/94 488 | 0.89 (0.87, 0.90) | 8598/37 448 | 0.93 (0.89, 0.97) |
| D5 | 25 010/105 987 | 0.86 (0.85, 0.88) | 9499/41 624 | 0.91 (0.88, 0.95) |
| D6 | 19 198/85 001 | 0.82 (0.81, 0.84) | 7365/33 916 | 0.87 (0.83, 0.91) |
| D7 | 22 263/100 627 | 0.80 (0.79, 0.82) | 8288/39 490 | 0.86 (0.82, 0.90) |
| D8 | 20 642/100 446 | 0.75 (0.73, 0.76) | 7851/39 850 | 0.83 (0.79, 0.87) |
| D9 | 18 688/94 375 | 0.72 (0.70, 0.73) | 6799/36 309 | 0.81 (0.77, 0.86) |
| D10 | 18 107/99 020 | 0.66 (0.65, 0.67) | 6830/38 458 | 0.78 (0.74, 0.83) |
| CI = confidence interval. D = decile. HR = hazard ratio. The models were adjusted for age at conscription, year of conscription, body mass index, IQ, parental education, and parental income. | | | | |

| **Supplemental table 17. Hazard ratios for depression diagnosis and dispensation of antidepressive medications by deciles of cardiorespiratory fitness in cohort and sibling analysis using complete-case analysis versus using multiple imputation.** | | | | | | | |  |
| --- | --- | --- | --- | --- | --- | --- | --- | --- |
| **Depression diagnosis** | | | | | | | |  |
| **Cohort analysis** | | | | **Sibling analysis** | | | |  |
|  | **Complete-case analysis (N=1 013 885)** |  | **Multiple imputation using chained equations**  **(N=1 249 130, K=20)** |  | **Complete-case analysis**  **(N=410 198)** |  | **Multiple imputation using chained equations**  **(N=524 525, K=20)** |  |
| **Deciles of fitness** | **HR (95% CI)** |  | **HR (95% CI)** | **Deciles of fitness** | **HR (95% CI)** |  | **HR (95% CI)** |  |
| D1 | Ref. |  | Ref. | D1 | Ref. |  | Ref. |  |
| D2 | 0.91 (0.88, 0.95) |  | 0.90 (0.87, 0.93) | D2 | 0.89 (0.82, 0.97) |  | 0.92 (0.85, 0.99) |  |
| D3 | 0.89 (0.86, 0.93) |  | 0.88 (0.85, 0.91) | D3 | 0.87 (0.80, 0.94) |  | 0.89 (0.82, 0.96) |  |
| D4 | 0.84 (0.81, 0.88) |  | 0.82 (0.79, 0.85) | D4 | 0.89 (0.81, 0.97) |  | 0.86 (0.79, 0.83) |  |
| D5 | 0.79 (0.76, 0.82) |  | 0.78 (0.75, 0.80) | D5 | 0.82 (0.75, 0.90) |  | 0.81 (0.75, 0.88) |  |
| D6 | 0.76 (0.73, 0.79) |  | 0.75 (0.73, 0.78) | D6 | 0.79 (0.71, 0.86) |  | 0.80 (0.73, 0.87) |  |
| D7 | 0.74 (0.71, 0.77) |  | 0.72 (0.69, 0.74) | D7 | 0.81 (0.73, 0.89) |  | 0.82 (0.75, 0.89) |  |
| D8 | 0.68 (0.65, 0.71) |  | 0.65 (0.63, 0.68) | D8 | 0.71 (0.64, 0.78) |  | 0.71 (0.65, 0.78) |  |
| D9 | 0.61 (0.59, 0.64) |  | 0.58 (0.56, 0.60) | D9 | 0.70 (0.63, 0.78) |  | 0.69 (0.63, 0.76) |  |
| D10 | 0.54 (0.52, 0.57) |  | 0.51 (0.49, 0.53) | D10 | 0.67 (0.59, 0.75) |  | 0.63 (0.57, 0.70) |  |
| **Dispensation of antidepressive medications** | | | | | | | |  |
| **Cohort analysis** | | | | **Sibling analysis** | | | |  |
|  | **Complete-case analysis (N=1 013 885)** |  | **Multiple imputation using chained equations**  **(N=1 249 130, K=20)** |  | **Complete-case analysis**  **(N=410 198)** |  | **Multiple imputation using chained equations**  **(N=524 525, K=20)** |  |
| **Deciles of fitness** | **HR (95% CI)** |  | **HR (95% CI)** | **Deciles of fitness** | **HR (95% CI)** |  | **HR (95% CI)** |  |
| D1 | Ref. |  | Ref. | D1 | Ref. |  | Ref. |  |
| D2 | 0.92 (0.90, 0.93) |  | 0.91 (0.90, 0.93) | D2 | 0.94 (0.91, 0.98) |  | 0.96 (0.92, 0.99) |  |
| D3 | 0.92 (0.90, 0.93) |  | 0.91 (0.89, 0.92) | D3 | 0.93 (0.90, 0.97) |  | 0.95 (0.92, 0.99) |  |
| D4 | 0.87 (0.85, 0.88) |  | 0.86 (0.84, 0.87) | D4 | 0.91 (0.88, 0.95) |  | 0.91 (0.88, 0.94) |  |
| D5 | 0.84 (0.82, 0.85) |  | 0.82 (0.81, 0.84) | D5 | 0.89 (0.86, 0.93) |  | 0.89 (0.85, 0.92) |  |
| D6 | 0.80 (0.78, 0.81) |  | 0.80 (0.78, 0.81) | D6 | 0.85 (0.82, 0.89) |  | 0.86 (0.83, 0.89) |  |
| D7 | 0.77 (0.76, 0.79) |  | 0.76 (0.75, 0.77) | D7 | 0.85 (0.81, 0.88) |  | 0.85 (0.82, 0.88) |  |
| D8 | 0.72 (0.70, 0.73) |  | 0.70 (0.69, 0.72) | D8 | 0.81 (0.78, 0.85) |  | 0.80 (0.77, 0.83) |  |
| D9 | 0.69 (0.68, 0.70) |  | 0.67 (0.66, 0.68) | D9 | 0.79 (0.76, 0.83) |  | 0.78 (0.74, 0.81) |  |
| D10 | 0.63 (0.62, 0.65) |  | 0.61 (0.60, 0.62) | D10 | 0.76 (0.72, 0.80) |  | 0.74 (0.71, 0.78) |  |
| CI = confidence interval. D = decile. HR = hazard ratio. ^a^The procedure was performed separately for the total cohort and full siblings (using K=20 repetitions), using linear and multinomial logistic models for continuous (fitness, BMI, and IQ) and categorical parameters (parental education and parental income). Age at conscription, conscription year, follow-up time, and censoring were used as complete auxiliaries, while weight and length at conscription were treated as partially observed auxiliaries (imputed using a linear model). | | | | | | | |  |
